# Mg^2+^ influx mediated by TRPM7 triggers the initiation of muscle stem cell activation

**DOI:** 10.1126/sciadv.adu0601

**Published:** 2025-04-04

**Authors:** Kotaro Hirano, Chika Nakabayashi, Mao Sasaki, Miki Suzuki, Yuta Aoyagi, Kaori Tanaka, Akira Murakami, Masaki Tsuchiya, Eiji Umemoto, Shuji Takabayashi, Yasuo Kitajima, Yusuke Ono, Takehisa Matsukawa, Masayuki Matsushita, Yasuyuki Ohkawa, Yasuo Mori, Yuji Hara

**Affiliations:** ^1^ School of Pharmaceutical Sciences, University of Shizuoka, Shizuoka 422-8526, Japan.; ^2^ Faculty of Pharmacy, Laboratory of Hygienic Chemistry, Juntendo University, Chiba 279-0013, Japan.; ^3^ Department of Medicine and Bioregulatory Science, Graduate School of Medical Sciences, Kyushu University, Fukuoka 812-8582, Japan.; ^4^ PRESTO, JST, Kawaguchi, Saitama 332-0012, Japan.; ^5^ Institute of Photonics Medicine, Hamamatsu University School of Medicine, Shizuoka 431-3192, Japan.; ^6^ Department of Immunology, Graduate School of Biomedical and Health Sciences, Hiroshima University, Hiroshima 734-8551, Japan.; ^7^ Department of Muscle Development and Regeneration, Institute of Molecular Embryology and Genetics, Kumamoto University, Kumamoto 860-0811, Japan.; ^8^ Department of Molecular and Cellular Physiology, Graduate School of Medicine, University of the Ryukyus, Okinawa 903-0213, Japan.; ^9^ Department of Synthetic Chemistry and Biological Chemistry, Graduate School of Engineering, Kyoto University, Kyoto 615-8510, Japan.

## Abstract

Muscle satellite cells (MuSCs) respond immediately to environmental cues upon skeletal muscle injuries. Despite decades of research into muscle regeneration, the specific molecular factors that trigger the transition of MuSCs from a quiescent to an active state remain largely unidentified. Here, we identify transient receptor potential melastatin 7 (TRPM7), an Mg^2+^-permeable ion channel, as a critical regulator of MuSC activation. *Trpm7* deletion in MuSCs reduced Mg^2+^ influx, impairing myofiber regeneration and leading to decreased MuSC numbers and cell cycle arrest during regeneration. These changes were linked to disrupted mTOR signaling, which drives the transition of MuSCs from G_0_ to G_Alert_ phase. In addition, *Trpm7*-deficient MuSCs exhibited impaired early responses, including quiescent projection retraction and AP-1 induction. Mg^2+^ supplementation rescued these defects, restoring normal MuSC activation. Our findings reveal a previously unrecognized mechanism where Mg^2+^ permeation through TRPM7 is essential for MuSC activation and efficient skeletal muscle regeneration, highlighting TRPM7 as a critical regulator of muscle repair.

## Teaser

Mg^2+^ permeation via the TRPM7 ion channel acts as a primary trigger for muscle stem cell activation and muscle regeneration.

## INTRODUCTION

Magnesium ion (Mg^2+^), recognized as the most abundant divalent cation in mammals, is essential for various cellular functions and responsible for more than 600 enzymatic reactions (*1*, *2*). The concentration gradient of Mg^2+^ between the intracellular and extracellular environment is infinitesimally small, with most intracellular Mg^2+^ (10 to 30 mM) bound to adenosine 5′-triphosphate (ATP) or cytoplasmic proteins and around 1 mM remaining unbound (*3*). Cytosolic Mg^2+^ dynamics is regulated by plasma membrane-resident ion channels/transporters including transient receptor potential (TRP) melastatin 7 (TRPM7), magnesium transporter 1 (MagT1), solute carrier family 41 member 1 (SLC41A1), TRPM6 (a homolog of TRPM7), Golgi-resident membrane magnesium transporter 1 (MMGT1), and the mitochondrial Mg^2+^ channel—mitochondrial RNA splicing 2 protein (Mrs2) (*4*). Despite the significance of Mg^2+^ in muscle physiology, such as regulation of muscle contraction (*2*), the potential role during muscle regeneration has not yet been investigated.

TRPM7 is a member of the TRPM subgroup of TRP channel superfamily and is a ubiquitously expressed bifunctional protein containing a kinase domain fused to the C terminus region (*5*, *6*). Systemic knockout mouse of *Trpm7* leads to embryonic lethality at embryonic day 7.5 (*7*). As an ion channel, TRPM7 elicits a small inward current at physiological membrane potentials and regulates cellular Ca^2+^, Mg^2+^, and Zn^2+^ levels to promote cellular function (*5*). TRPM7 can transduce Mg^2+^ to maintain Mg^2+^ homeostasis (*8*–*11*), while free Mg^2+^ or intracellular Mg-ATP is known to inhibit TRPM7 currents (*8*). As a kinase, the kinase domain of TRPM7 belongs to a family of atypical protein kinases and is reported to phosphorylate several substrates including annexin A1 (*12*), phospholipase C γ2 (*13*), eukaryotic elongation factor 2 kinase (*14*), and TRPM7 itself (*15*). TRPM7 is also reportedly activated by changes in Mg-ATP concentration (*8*), reactive oxygen species (*16*), mechanical stress (*17*), and alkalization (*18*). However, the physiological role of TRPM7 in skeletal muscle regeneration remains unclear.

Skeletal muscle regeneration is accomplished by muscle-resident stem cells known as muscle satellite cells (MuSCs). Upon injury, MuSCs are activated to exit quiescence and enter the cell cycle (*19*) to proliferate and differentiate into myoblasts and then fuse to become regenerating myofibers (*20*). During this regenerative process, collapse of the microenvironment (niche) activates MuSCs with marked change in cellular composition (*21*). Several cellular responses are thought to rapidly arise during the transition from MuSC quiescence to an activated state; metabolic switching, transcriptional and translational regulation, and morphological changes (*22*–*25*). One of the earliest responses, recently found, is the retraction of neuronal-like projections (quiescent projections) in MuSCs (*26*) via small guanosine triphosphatase (GTPase) signaling pathways, which regulate the cytoskeletal architecture of MuSCs. In turn, fundamental stress response pathways are up-regulated. Within hours after muscle injury, mitogen-activated protein kinase (MAPK p44/42) is activated to induce the transcriptional family of activator protein-1 (AP-1) genes, which are hallmarks of the cellular stress response (*25*, *27*, *28*). Despite the fact that immediate cellular responses to external stimuli are due to an influx of divalent ions (*29*, *30*), whether ion channels regulate these early events in MuSCs has not yet been reported.

In this study, we show in a tissue-resident stem cell that Mg^2+^ incorporation via TRPM7 is a main trigger for the quiescent-to-activation transition. Our results demonstrate that MuSC-specific *Trpm7* conditional knockout (*Trpm7* cKO) mice exhibit impaired muscle regeneration following muscle injury, owing to impaired activation of MuSCs. We also show that TRPM7 promotes the mammalian target of rapamycin (mTOR) signaling pathway via Mg^2+^ influx in MuSCs during cell cycle entry. Furthermore, TRPM7 is required for MuSCs to enter the G_Alert_ phase, a distinct quiescent state from G_0_ (*31*), by activating the mTOR pathway. Moreover, TRPM7 is responsible for early activation of MuSCs by promoting retraction of quiescent projections and the regulation of AP-1 genes. Our study reveals a mechanism underlying muscle regeneration: Mg^2+^ influx via TRPM7 is an initial event in MuSC activation that is essential for skeletal muscle homeostasis.

## RESULTS

### Magnesium ion dynamics in MuSCs during skeletal muscle regeneration

**Fig. 1.**
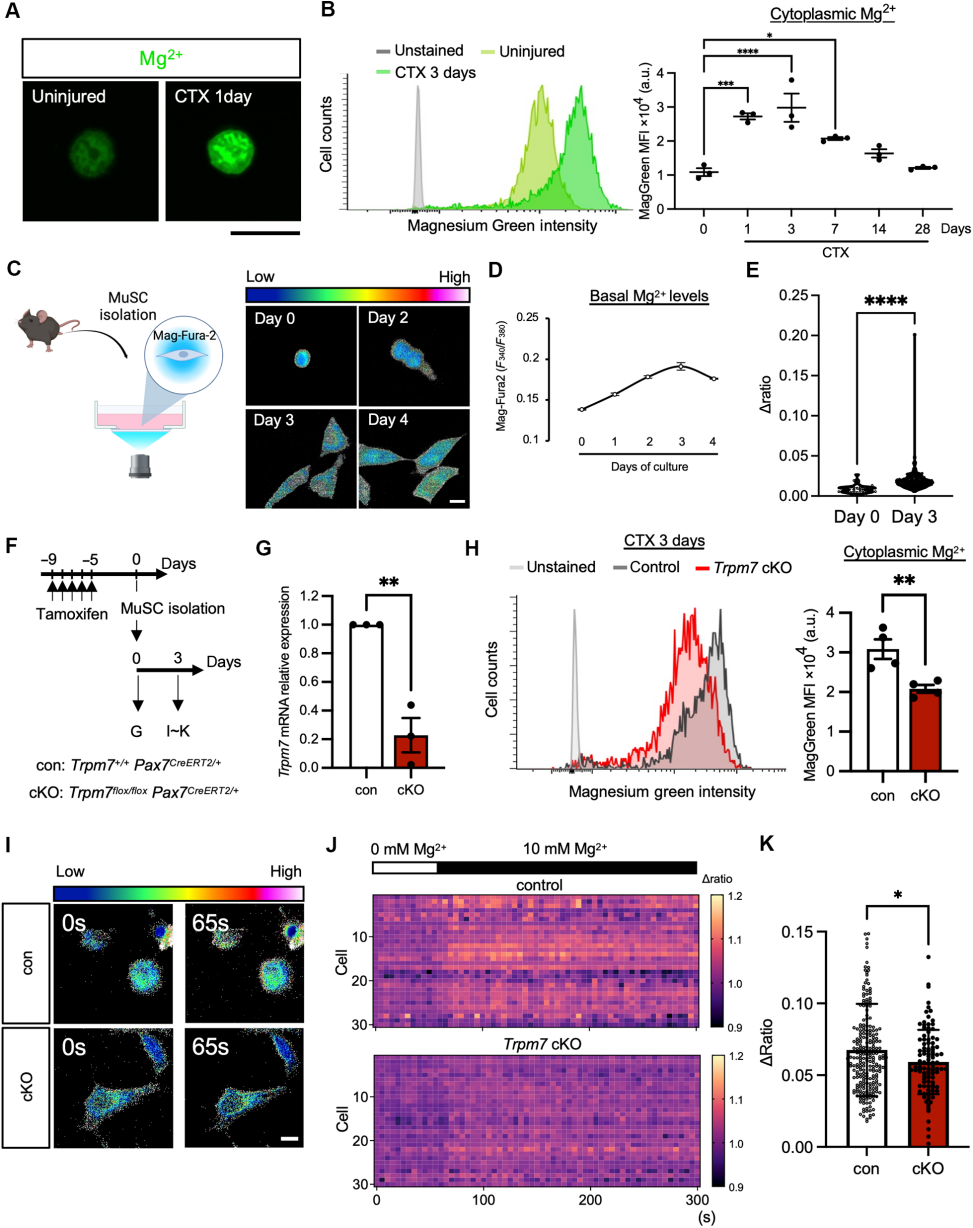
Magnesium ion dynamics in MuSCs during skeletal muscle regeneration. (**A** and **B**) Mg^2+^ measurements in MuSCs using Magnesium Green, AM. (A) Representative fluorescent images of MuSCs from uninjured (left) and injured (1 day after injury) (right) muscle. (B) Left: Histograms of FACS data. Gray: unstained MuSCs; light green: uninjured MuSCs; dark green: injured MuSCs 3 days post-CTX. Right: Quantification of mean fluorescence intensity at indicated time points. (**C** to **E**) Mg^2+^ measurements using Mag-Fura-2 (C) Left: Schematic of Mg^2+^ imaging. Right: Fluorescent images of MuSCs cultured for 0 to 4 days. Look-up tables (LUTs) indicate signal intensity. (D) Quantification of basal Mg^2+^ levels in MuSCs cultured for 0 to 4 days. (E) Maximum fluorescent intensity (Δratio) of Mag-Fura-2 after addition of 10 mM Mg^2+^ (final concentration) subtracted by the mean basal intensity. (**F**) Time course for induction of *Trpm7* deficiency. *Trpm7^+/+^*; *Pax7^CreERT2/+^* was used as control (con) and *Trpm7^flox/flox^*; *Pax7^CreERT2/+^* as knockout (cKO). (**G**) *Trpm7* mRNA expression in freshly isolated MuSCs. (**H**) Cytosolic Mg^2+^ measurements of MuSCs 3 days post-CTX injury. Left: Histogram of flow cytometric data using Magnesium green. Light gray: Unstained MuSCs; dark gray: con MuSCs; and red: cKO MuSCs. Right: Quantification of increase in cytosolic Mg^2+^. (**I** to **K**) Mg^2+^ imaging of MuSCs cultured for 3 days. (I) Fluorescent images of control and cKO MuSCs before (0 s) and after (65 s) adding 10 mM Mg^2+^. (J) Heatmap showing Δ*F*/*F* of Mag-Fura-2 transients in pseudo-color (30 cells per group). (K) Δratio of Mag-Fura-2 in con versus cKO MuSCs (control: *n* = 241 cells, cKO: *n* = 101 cells, three mice per condition). Scale bars, 10 μm in [(A), (C), and (I)]. a.u, arbitrary unit.

Despite the importance of Mg^2+^ being highlighted in mammals (*2*, *3*), its function and dynamics in tissue-resident stem cells remains poorly understood. To investigate the function of Mg^2+^ in MuSCs during skeletal muscle regeneration, we used a cell-permeable Mg^2+^ indicator, Magnesium Green, to measure cytosolic Mg^2+^ levels in MuSCs. We detected mean fluorescence intensity by flow cytometry during skeletal muscle regeneration at 1, 3, 7, 14, 21, and 28 days post-injury. Cytosolic Mg^2+^ elevated in activated MuSCs at 1 or 3 days after regeneration compared with uninjured MuSCs (Fig. 1, A and B, and fig. S1A). Cytosolic Mg^2+^ levels gradually decreased at 7 to 14 days after injury and returned to resting levels by 28 days after injury when a large population of MuSCs returns to quiescence (Fig. 1B and fig. S1B) (*32*). We further investigated the dynamics of Mg^2+^ using a ratiometric Mg^2+^ indicator, Mag-Fura-2, on an ex vivo platform (*33*). After isolating MuSCs and inducing activation in culture, Mg^2+^ incorporation was increased in activated or proliferating MuSCs (Fig. 1, C to E). These results reveal that MuSCs undergo notable changes in Mg^2+^ dynamics during skeletal muscle regeneration.

### Magnesium ion influx is mediated by TRPM7 in MuSCs

Intracellular Mg^2+^ dynamics is tightly regulated by several ion channels and transporters. In silico analysis found that prominent genes responsible for Mg^2+^ transport (such as *Trpm7* and *Slc41a*) are highly expressed in freshly isolated MuSCs (fig. S1C). We also found that *Trpm7*, but not *Trpm6*, was highly expressed in MuSCs (fig. S1C). To detect TRPM7 at the protein level, immunofluorescent analysis was performed in freshly isolated MuSCs (fig. S1D), cultured MuSCs (fig. S1E), and differentiated MuSCs (fig. S1F). TRPM7 expression was observed at the plasma membrane at each stage of myogenesis, suggesting that TRPM7 is involved throughout the myogenic process. To determine the function of TRPM7 in MuSCs, we generated MuSC-specific *Trpm7* cKO mice by crossing *Pax7^CreERT2/+^* mice (*34*) with *Trpm7*-floxed mice (fig. S1G). *Trpm7* genetic ablation was induced by intraperitoneal injection of tamoxifen (TMX) in *Trpm7^flox/flox^*; *Pax7^CreERT2/+^* mice (Fig. 1F). Genetic analysis confirmed the gene deletion of *Trpm7* (Fig. 1G and fig. S1H).

To explore whether TRPM7 acts as a Mg^2+^-permeable ion channel in MuSCs, we measured cytosolic Mg^2+^ levels in MuSCs loaded with Magnesium Green at 3 days post-muscle injury. Cytosolic Mg^2+^ levels declined in MuSCs isolated from *Trpm7* cKO mice (Fig. 1H), indicating that TRPM7 is an ion channel required for Mg^2+^ permeability in MuSCs during muscle regeneration. Next, we isolated and cultured MuSCs from *Trpm7* cKO mice and measured Mg^2+^ incorporation using Mag-Fura-2. We observed a rapid increase of intracellular Mg^2+^ levels after addition of Mg^2+^ in control MuSCs (Fig. 1, I and J). However, intracellular Mg^2+^ influx in *Trpm7* cKO MuSCs was declined compared with controls (Fig. 1, I to K).

### TRPM7 is critical for muscle regeneration in vivo

**Fig. 2.**
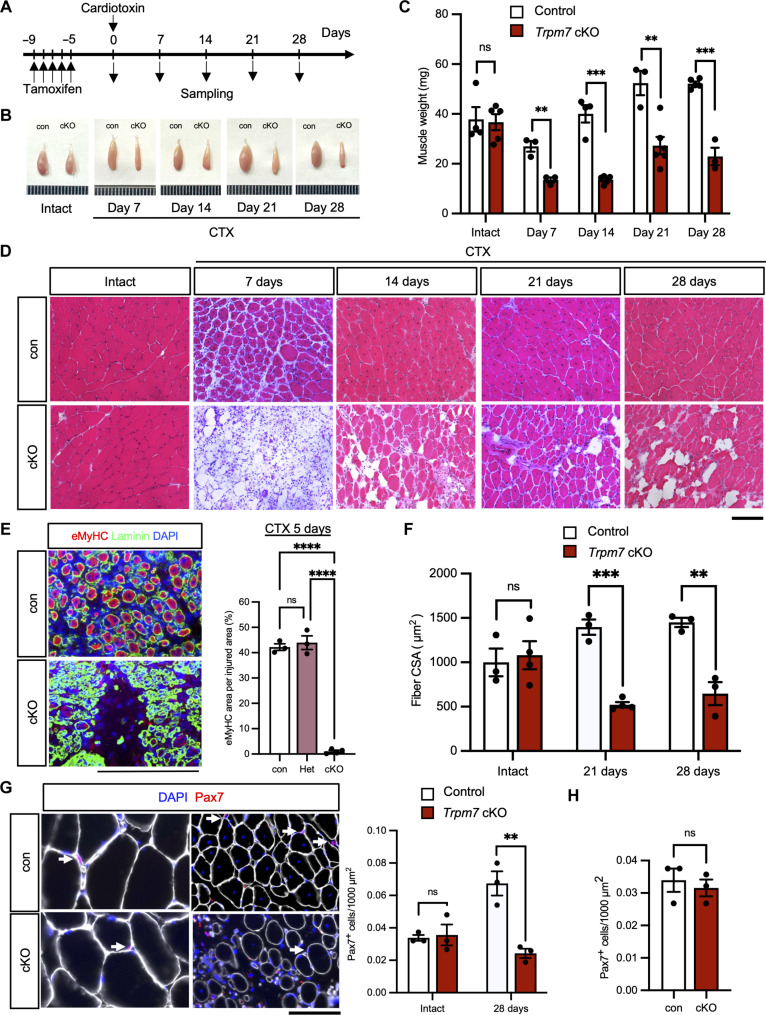
Impaired regeneration capacity of MuSC-specific *Trpm7*-deficient muscle after injury. (**A**) Time course for induction of *Trpm7* deficiency, injection of TA with CTX, and isolation of regenerating muscle samples for [(B) to (D)]. (**B**) Representative images of regenerating TA muscle samples isolated from con (left) and cKO mice (right). (**C**) Weight of TA muscle samples after CTX-induced muscle injury (*N* = 3 to 5 mice per condition). (**D**) Hematoxylin and eosin staining of cross sections from intact and CTX-injected TA muscle samples harvested at indicated time points. Top panels: con; bottom panels: cKO mice. (**E**) Detection of eMyHC in cross sections from control (top) and cKO muscle (bottom). The area of eMyHC per section was evaluated 5 days after CTX treatment. (*N* = 3 to 4 mice per condition). (**F**) Cross-section area (CSA) of intact and regenerating myofibers at 21 and 28 days post-CTX injection (*N* = 3 mice per condition). (**G**) Number of Pax7-positive cells in intact and regenerating myofibers at 28 days post-CTX injection. Representative images of intact and regenerating muscle sections. White arrows indicate Pax7-positive cells (*N* = 3 mice per condition). (**H**) Quantification of MuSCs on muscle section, 1 month following the first TMX injection. Representative immunohistochemical images of TA muscle sections are shown in fig S1I. Pax7^+^ cells per 1000 μm^2^. Scale bars, 100 μm in [(D), (E), and (G)].

To determine whether TRPM7 is involved in muscle regeneration, cardiotoxin (CTX) was injected into tibialis anterior (TA) muscle, and resultant muscle samples were collected at indicated time points (Fig. 2A). TA muscle samples collected from *Trpm7* cKO mice were smaller and weighed significantly less compared with control mice (Fig. 2, B and C, and fig. S2A). Correspondingly, the histological analysis of *Trpm7* cKO mice showed significant defects in muscle regeneration (Fig. 2D). Next, we performed immunohistochemical analysis on CTX-injected muscle samples. Embryonic myosin heavy chain (eMyHC), a marker of regenerating myofibers, was detected in CTX-injected muscles. The area of eMyHC-positive myofibers decreased in *Trpm7* cKO mice, suggesting that fewer newly formed regenerating myofibers were generated in *Trpm7* cKO mice (Fig. 2E). Also, by genetically labeling MuSCs using *Pax7^CreERT2/+^*; *Rosa26^YFP/+^* mice and administering CTX, *Trpm7* cKO mice showed limited newly formed yellow fluorescent protein (YFP)–positive myofibers (fig. S2E). In support of this, *Trpm7* cKO mice showed increased fibrosis (fig. S2F) and decreased cross-sectional myofiber areas (Fig. 2F and fig. S2, B to D). Last, we examined the number of Pax7-positive cells in intact and injured muscle sections. While the number of Pax7-positive cells before injury was comparable between groups, a significant decline was observed in *Trpm7* cKO mice at 28 days post-injury (Fig. 2G). These results suggest that TRPM7 is essential for muscle regeneration and the maintenance of the MuSC pool.

Next, we examined whether the kinase activity of TRPM7 is also important for muscle regeneration. TRPM7^K1646R^ knock-in mice (*35*), a kinase-inactive mutant mouse model (TRPM7-KR mice), were injected with CTX for muscle injury. No anatomical differences were observed in regenerating TA muscles from TRPM7-KR mice compared with control mice (fig. S3A). Also, eMyHC-positive area (fig. S3B) and cross-sectional myofiber areas (fig. S3C) in TRPM7-KR mice were comparable with control mice. These results indicate that TRPM7’s ion channel activity is more important than the kinase activity during muscle regeneration.

We also investigated the effect of *Trpm7* deficiency in skeletal muscle. The deletion of *Trpm7* was induced by administering TMX to mice, followed for 1 month after *Cre* induction. There was no change in body weight, muscle weight, or anatomy at 1-month post-TMX administration. There were also no obvious changes in the number of sublaminar MuSCs in skeletal muscle sections (Fig. 2H and fig. S1I) or MuSCs from isolated myofibers (fig. S1J) at 1 month after TMX administration. This indicates that *Trpm7* gene disruption does not perturb MuSCs in intact skeletal muscle.

### TRPM7 is essential for myogenesis and cell cycle regulation

**Fig. 3.**
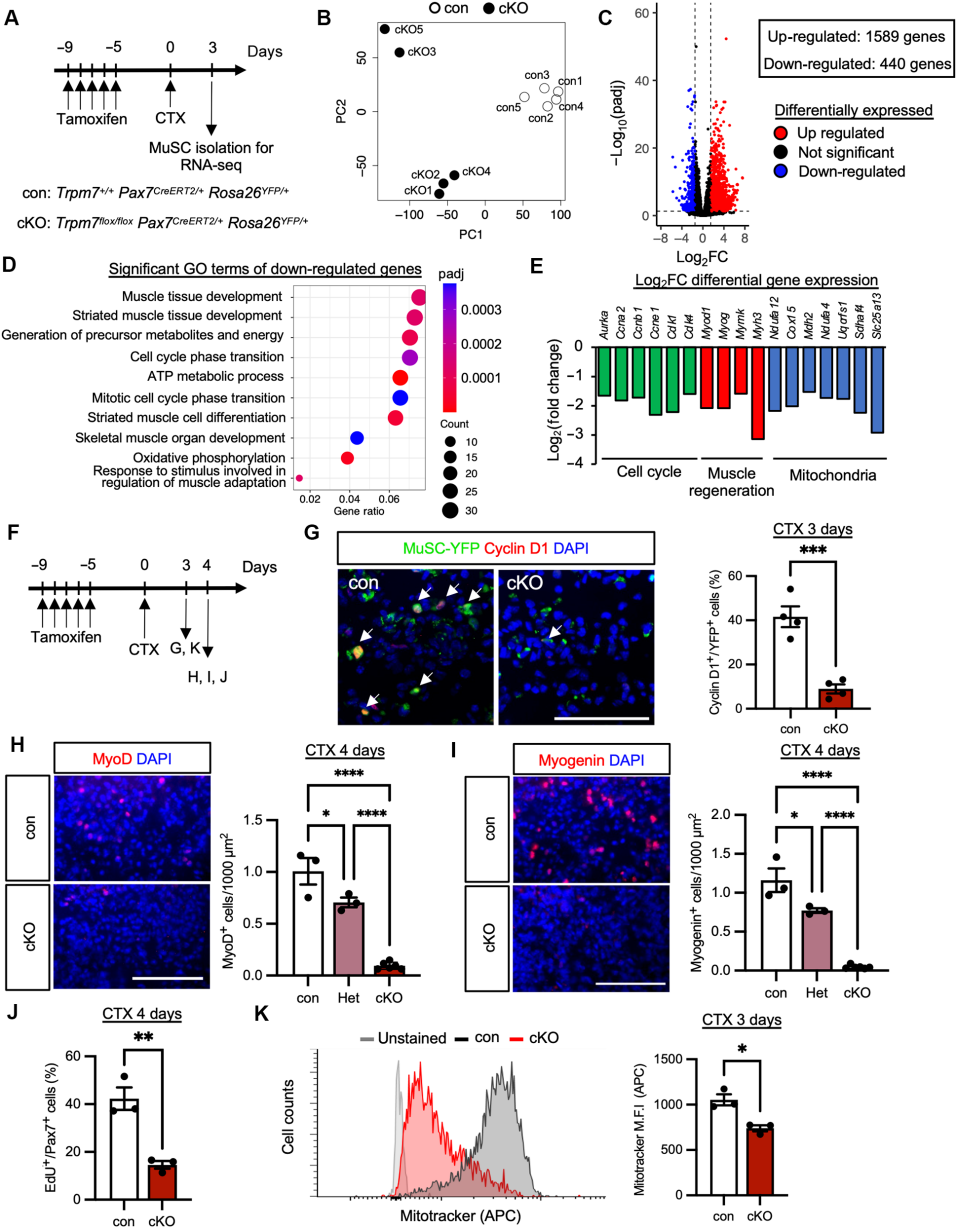
Characterization of *Trpm7*-deficient MuSCs based on RNA-seq analysis. (**A**) Time course for induction of *Trpm7* deficiency, muscle injury, and sampling of MuSCs for RNA-seq. (**B**) Principal components analysis (PCA) of RNA-seq data. (**C**) Volcano plot of differentially expressed genes. Red and blue dots represent the genes with significantly increased (up-regulated) or decreased (down-regulated) expression, respectively (padj < 0.05). (**D**) GO enrichment analysis of significantly down-regulated genes in cKO. (**E**) Log_2_fold changes (Log_2_FCs) of representative down-regulated genes in cKO. (**F**) Time course for induction of *Trpm7* deficiency, muscle injury, and sampling for [(G) to (K)]. (**G**) Detection of YFP-positive MuSCs and Cyclin D1 in cross sections from control (left) and cKO muscle (right). White arrows indicate YFP and Cyclin D1 double-positive cells. The ratio of Cyclin D1 per YFP-positive MuSCs was evaluated 3 days after CTX treatment (*N* = 4 mice per condition). (**H**) Detection of MyoD-positive cells in cross sections from con (top) and cKO muscle (bottom). MyoD-positive cells per 1000 μm^2^ were evaluated 4 days after CTX treatment (*N* = 3 to 6 mice per condition). (**I**) Detection of Myogenin-positive cells in cross sections from control (top) and cKO muscle (bottom). Myogenin-positive cells per 1000 μm^2^ were evaluated 4 days after CTX treatment (*N* = 3 to 6 mice per condition). (**J**) Ratio of EdU-positive cells per Pax7-positive cells were evaluated 4 days after CTX treatment (*N* = 3 mice per condition). (**K**) Representative FACS profiles of MuSCs isolated at 3 days post-injury, stained with MitoTracker Deep Red. Left: Histograms representing the mean fluorescent intensity of MitoTracker. Light gray: Unstained MuSCs; dark gray: con MuSCs; and red histogram: cKO MuSCs isolated from injured muscle 3 days post-CTX injection. Right: Quantification of mean fluorescence intensity. Scale bars, 100 μm in [(G) to (I)] and 10 μm (L).

To gain a global understanding of the factors leading to impaired muscle regeneration in *Trpm7* cKO mice, we performed RNA sequencing (RNA-seq) on MuSCs isolated at 3 days post-CTX injury (Fig. 3A and fig. S4A). Principle components analysis showed separate clusters for control and *Trpm7* cKO MuSCs (Fig. 3B). Nearly 2000 genes were differentially expressed (Fig. 3C and fig. S4B), and Gene Ontology (GO) terms of differentially down-regulated genes were related to cell cycle, myogenesis, and mitochondria (Fig. 3D and fig. S4C). These genes were notable cell cycle regulators (*Cdk1* and *Cdk4*), master regulators of myogenesis (*MyoD* and *Myog*), and main components of mitochondria (*Ndufa12* and *Cox15*). This provides evidence that TRPM7 functions upstream of major factors in MuSCs during muscle regeneration.

On the basis of these results, immunofluorescent analysis was performed to detect cell cycle related maker (Cyclin D1) and myogenic regulators (MyoD and myogenin) in CTX-injured muscle isolated at 3 or 4 days post-injury (Fig. 3F). Cyclin D1 together with myogenic regulating factors were decreased in *Trpm7* cKO mice (Fig. 3, G to I). In contrast, TRPM7-KR mice showed the normal expression of myogenic regulating factors 4 days post-injury (fig. S3, D and E). To examine the rate of S phase entry of myogenic cells during regeneration at 3 or 4 days post-injury, 5-ethynyl-2′-deoxyuridine (EdU) incorporation assays were performed. The number of EdU-positive MuSCs decreased in *Trpm7* cKO mice (Fig. 3J and fig. S4E). In addition, Ki67, a maker of cell cycle entry, was also detected at 3 days post-injury (fig. S4D). The number of M-cadherin– and Ki67 double-positive cells (i.e., myogenic cells that had entered the cell cycle) was decreased in *Trpm7* cKO mice (fig. S4F). These data indicate that *Trpm7* deficiency causes defects in cell cycle entry of MuSCs, leading to reduced proliferative ability during muscle regeneration.

Next, we investigated whether *Trpm7* deletion results in mitochondrial defects, as mitochondria-related genes were significantly decreased in our RNA-seq analysis (Fig. 3E). Muscle regeneration was induced by CTX injury, and isolated MuSCs were stained with a mitochondria dye, MitoTracker Deep Red, and then analyzed by fluorescence-activated cell sorting (FACS). Mean fluorescence intensity significantly decreased in MuSCs isolated from *Trpm7* cKO mice compared with controls (Fig. 3K), indicating that TRPM7 is essential for mitochondrial homeostasis in MuSCs.

### TRPM7 deletion impairs MuSCs activation and proliferation

**Fig. 4.**
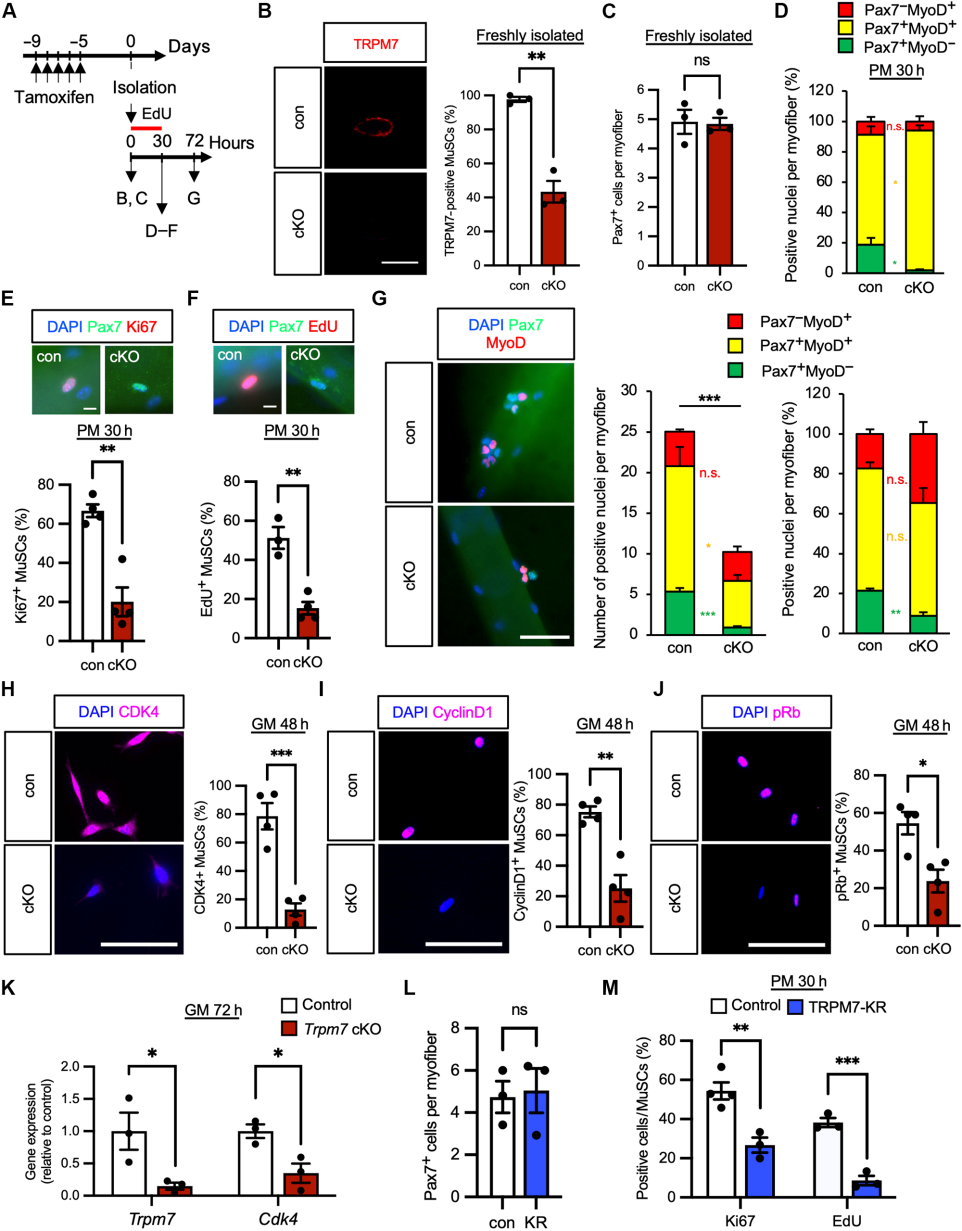
*Trpm7* deficiency inhibits cell cycle entry and proliferation of MuSC. (**A**) Time course for induction of *Trpm7* deficiency, harvesting of myofibers, and EdU incorporation. (**B** and **C**) TRPM7 and Pax7 expression in freshly isolated myofibers. (B) TRPM7 (red) detection in MuSCs; nuclei (DAPI, blue). Top: con MuSCs; bottom: cKO MuSCs. Right: Quantification of TRPM7-positive MuSCs (>50 MuSCs per condition, *N* = 3 mice). (C) Pax7-positive MuSCs per myofiber (>15 myofibers, *N* = 3 mice). (**D** to **F**) MuSC analysis after 30-hour culture. (D) Pax7 and MyoD expression (>15 myofibers, *N* = 3 mice). (E) Pax7 and Ki67 expression (>15 myofibers, *N* = 4 mice). (F) EdU incorporation assay in Pax7-positive MuSCs (>15 myofibers, *N* = 3 to 4 mice). (**G**) Immunofluorescent analysis after 72-hour culture. Left: Pax7 (green), MyoD (red), and nuclei (blue) in con (top) and cKO (bottom) MuSCs. Middle: Pax7- and MyoD-positive nuclei per myofiber. Right: Percent of positive nuclei (>15 myofibers, *N* = 5 mice). (**H** to **J**) Detection of CDK4-positive, Cyclin D1-positive, and phosphorylated Rb (pRb)–positive MuSCs cultured in growth medium for 48 hours. Left panels: CDK4 (H), Cyclin D1 (I), or pRb (J) were detected in con (top panels) and cKO (bottom panels). Right graphs: Quantification of CDK4 (H)–, Cyclin D1 (I)–, or pRb (J)–positive MuSCs. (>300 MuSCs per condition from *N* = 4 mice). (**K**) qPCR of MuSCs cultured 72 hours. (**L** and **M**) TRPM7-KR MuSCs. (L) Pax7-positive cells per myofiber (>15 myofibers, *N* = 3 mice). (M) Ki67 expression and EdU incorporation after 30 hours (>15 myofibers, *N* = 3 to 4 mice). Scale bars, 10 μm in [(B), (E), (F), (G), (H), (I), and (J)]. h, hours.

To investigate the impact of TRPM7 on MuSCs activation and proliferation, we analyzed them in floating myofibers, which have a well-preserved microenvironmental niche surrounding MuSCs. Extensor digitorum longus (EDL) muscles were isolated from mice treated with TMX for five consecutive days (Fig. 4A). Immunofluorescence analysis confirmed the effectiveness of *Trpm7* deficiency in freshly isolated MuSCs of myofibers (Fig. 4B). Under this condition, the number of quiescent MuSCs per myofiber was comparable in *Trpm7* cKO mice and controls (Fig. 4C), indicating that TRPM7 does not contribute to the maintenance of MuSCs. Next, isolated myofibers were cultured for 30 hours in medium, and the status of MuSCs was examined by detection of Pax7 and MyoD, transcription factors highly expressed in undifferentiated and activated MuSCs, respectively.

The proportion of Pax7-positive/MyoD-negative cells (i.e., nonactivated reserve cells) was decreased in *Trpm7* cKO mice compared with controls (Fig. 4D). Moreover, the number of Ki67-positive or EdU-positive MuSCs was also decreased in *Trpm7* cKO (Fig. 4, E and F). These data indicate that TRPM7 plays a role in maintaining the MuSC pool and cell cycle entry.

We further examined the status of MuSCs on myofibers cultured for 3 days. The total number of MuSCs and the proportion of Pax7-positive/MyoD-negative cells (i.e., self-renewing cells) significantly decreased in *Trpm7* cKO compared with control mice (Fig. 4G). This shows that TRPM7 is essential for MuSC self-renewal and proliferation. To evaluate proliferation ex vivo, EdU incorporation assay was also performed on isolated MuSCs (fig. S5D). After 72-hour culture in growth media, the number of EdU-positive MuSCs was reduced in *Trpm7* cKO mice (fig. S5E), consistent with our results on MuSCs cultured on myofibers. To determine whether cell death caused the reduction of MuSCs, TUNEL (terminal deoxynucleotidyl transferase–mediated deoxyuridine triphosphate nick end labeling) assay was performed. TUNEL-positive cells increased in *Trpm7* cKO MuSCs (fig. S5F), indicating that cell death partially takes part in the *Trpm7*-deficient phenotype during proliferation.

As *Trpm7* deficiency results in late cell cycle entry, we determined which cell cycle point is regulated by TRPM7 (fig. S5A). Cyclin-dependent kinase 4 (CDK4), Cyclin D1, and phosphorylated retinoblastoma protein (pRb)–positive MuSCs were reduced in *Trpm7* cKO MuSCs, indicating that TRPM7 is responsible for regulating this restriction point in MuSCs (Fig. 4, H to J, and fig. S5B). To support these data, we detected cell cycle regulating genes and found that *Cdk4* is down-regulated (Fig. 4K). In addition, levels of p21, a negative regulator of the cell cycle, were comparable in control and *Trpm7* cKO mice, indicating that TRPM7 directly up-regulates the formation of the Cyclin D–CDK4 complex (fig. S5C).

To examine whether the kinase activity of TRPM7 is also important in MuSCs, myofibers from TRPM7-KR mice were isolated and investigated. The number of freshly isolated MuSCs in myofibers was comparable between TRPM7-KR mice and controls (Fig. 4L). Next, isolated myofibers were cultured for 30 hours in plating medium. The number of Ki67-positive or EdU-positive MuSCs was visibly decreased in TRPM7-KR mice compared with controls (Fig. 4M). The proportion of Pax7-positive/MyoD-negative cells (i.e., self-renewing cells) was decreased in TRPM7-KR mice compared with control mice, while the total number of MuSCs was comparable in the both mouse lines (fig. S5, G and H). Moreover, we determined the capacity of *Trpm7*-deficient MuSCs to form myotubes (fig. S5I). Four days after induction of differentiation, the fusion index was significantly decreased by *Trpm7* deficiency (fig. S5J). In contrast, the fusion index was not affected by TRPM7 kinase inactivation (fig. S5K). Together, these data suggest that TRPM7 promotes cell cycle entry for activation, thus enabling MuSCs to proliferate for myogenesis. In addition, TRPM7 kinase activity may take part in the early part of MuSC cell cycle entry but not during proliferation.

### TRPM7 positively regulates the mTOR pathway during MuSC activation

**Fig. 5.**
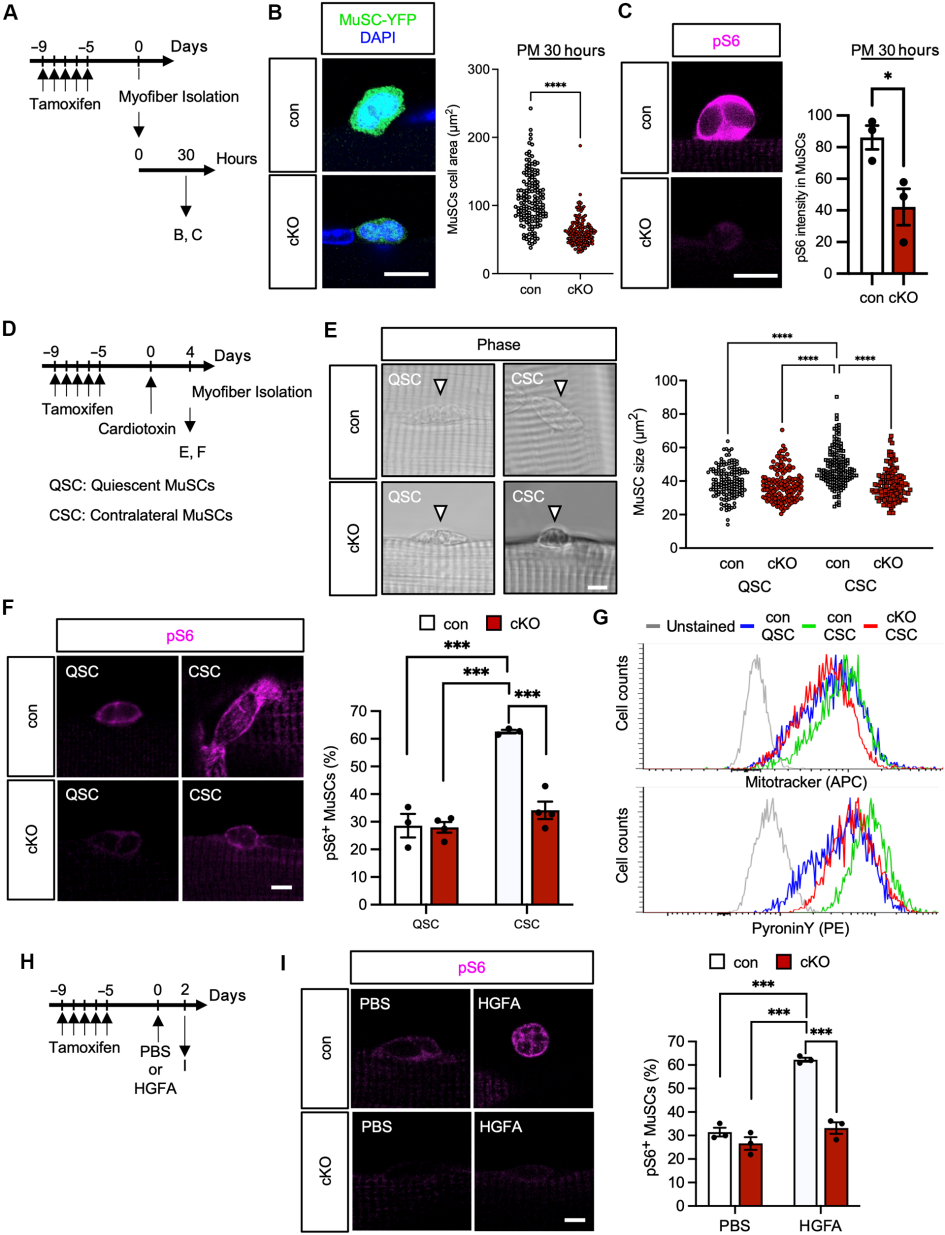
Loss of TRPM7 impairs activation of the mTOR signaling and induction of G_Alert_ in MuSCs. (**A**) Time course for induction of *Trpm7* deficiency and isolation of myofibers. (**B** and **C**) Analysis of MuSCs on myofibers cultured for 30 h. (B) MuSC size: Representative YFP-positive MuSC images and size distribution (>100 cells per condition). (C) pS6: Representative fluorescent images and quantification of pS6 intensity (>50 cells per condition, *N* = 3 mice). (**D**) Time course for induction of *Trpm7* deficiency, injection of CTX, and isolation of myofibers. MuSCs on myofibers without injury was defined as quiescent satellite cells (QSCs). MuSCs on myofibers isolated from the contralateral leg of injury was defined as contralateral satellite cells (CSCs). Myofibers were isolated 4 days after CTX injection for G_Alert_ induction. (**E**) Morphological evaluation of MuSCs after induction of G_Alert_. Phase contrast images of con (top) and cKO (bottom) MuSCs. Size distribution of QSCs and CSCs (>100 MuSCs per condition; data are individually plotted.). (**F**) pS6 in QSCs and CSCs: Representative images and quantification in con and cKO MuSCs (>50 cells per condition, *N* > 3 mice). (**G**) FACS histograms of MitoTracker and PyroninY staining. Gray: Unstained MuSCs; blue: con QSCs; green: con CSCs; red: cKO CSCs. (**H**) Time course for induction of *Trpm7* deficiency, injection of HGFA, and isolation of myofibers. MuSCs from HGFA-treated mice were defined as HGFA for G_Alert_ induction. PBS was used for control. (**I**) HGFA effect on pS6 in cKO MuSCs: Representative images and quantification of pS6 in con and cKO MuSCs after G_Alert_ induction with HGFA (>50 cells per condition, *N* = 3 mice). Scale bars, 10 μm in [(B), (C), (E), (F), and (I)].

The data so far provide evidence showing that TRPM7 is essential for cell cycle entry of MuSCs. We analyzed the status of MuSCs on myofibers after culturing for 30 hours (Fig. 5A).

There was a significant decrease in size of *Trpm7* cKO MuSCs compared with controls (Fig. 5B) showing that TRPM7 is essential for MuSC size growth after activation. TRPM7 is known to mediate various pathways, including the phosphoinositide 3-kinase/Akt/mTOR (PI3K/Akt/mTORC1) pathway (*36*) that is one of the earliest signaling pathways in MuSC activation (*37*). Therefore, we first detected the phosphorylated form of Akt as a substrate of PI3K (fig. S6A). The immunofluorescent intensity of phosphorylated Akt was decreased in *Trpm7*-deficient MuSCs compared with controls (fig. S6B). As downstream targets of mTORC1, we next examined the phosphorylation of S6 and eukaryotic translation initiation factor 4E binding protein 1 (4E-BP). Levels of phosphorylated forms of both S6 (pS6) and 4E-BP (p4E-BP) were decreased in *Trpm7*-deficient MuSCs (Fig. 5C and fig. S6D), while total S6 and 4E-BP protein levels were comparable (fig. S6, C and D). We further determined whether the mTOR pathway regulates cell cycle entry in MuSCs. MuSCs isolated from wild-type mice were cultured in the presence of mTOR pathway inhibitors: 4EGI (*38*), rapamycin (*39*), and INK128 (*40*) (fig. S6E). Treatment with these mTOR pathway inhibitors significantly reduced the ratio of Cyclin D1–positive MuSCs (fig. S6F). Together, these data indicate that TRPM7 acts as an upstream factor of the PI3K/Akt/mTORC1 pathway to promote cell cycle entry and activation of MuSCs.

### *Trpm7*-deficient MuSCs contralateral to injury display no G_Alert_ response

MuSCs exist in the mitotically quiescent stage (G_0_) during homeostasis, but upon muscle injury, they enter the G_1_ phase for activation. Accumulating evidence has shown that during the transition from G_0_ to G_1_ phase, MuSCs enter a potentially active phase (but still defined as a mitotically quiescent phase), which is known as the G_Alert_ phase (*41*). As transition to the G_Alert_ phase is controlled by mTORC1 signaling, we hypothesized that TRPM7 may regulate this transition. Hindlimbs on one side of the body were injured by CTX injection, and the contralateral myofibers were isolated (Fig. 5D). Consistent with previous studies in control mice, MuSC size and the number of pS6-positive cells increased in MuSCs on contralateral myofibers [these MuSCs are defined as contralateral satellite cells (CSCs)] compared to uninjured myofibers [or quiescent satellite cells (QSCs)] (Fig. 5, E and F). However, in *Trpm7* cKO mice, MuSC size and the number of pS6-positive cells were unchanged in CSCs compared with QSCs (Fig. 5, E and F). To precisely specify the function of TRPM7 in G_Alert_ induction, we measured mitochondrial mass and RNA levels in QSCs and CSCs. CSCs isolated from *Trpm7* cKO mice displayed decreased levels of MitoTracker staining and Pyronin Y (an RNA cationic dye) staining, indicating that TRPM7 is essential for G_Alert_ progression in MuSCs (Fig. 5G). Moreover, to examine the role of TRPM7 in the G_Alert_ transition, hepatocyte growth factor (HGF) activator (HGFA) was administered by tail vein injection to induce G_Alert_ in MuSCs (*42*) (Fig. 5H). Consistent with our results in the CTX injury model, the number of pS6-positive cells was unchanged in *Trpm7* cKO mice following HGFA induction compared with phosphate-buffered saline (PBS) administration (Fig. 5I). These results indicate that TRPM7 is involved in the transition from G_0_ to G_Alert_ phase by activation of the mTOR signaling cascade.

MuSCs that enter G_Alert_ are prone to faster activation than QSCs. Isolated myofibers from control or *Trpm7* cKO mice were cultured for 24 hours to induce MuSC activation. The ratio of Ki67-positive/Pax7-positive cells was significantly lower in both *Trpm7*-deficient QSCs and CSCs compared with controls but was slightly increased in *Trpm7*-deficient CSCs compared with QSCs (fig. S6G). Together, these results indicate that deletion of *Trpm7* in MuSCs leads to impaired transition from the G_0_ to G_Alert_ phase, which will ultimately affect the ability of MuSCs to undergo muscle regeneration.

### TRPM7 is responsible for quiescent to activation transition of MuSCs

**Fig. 6.**
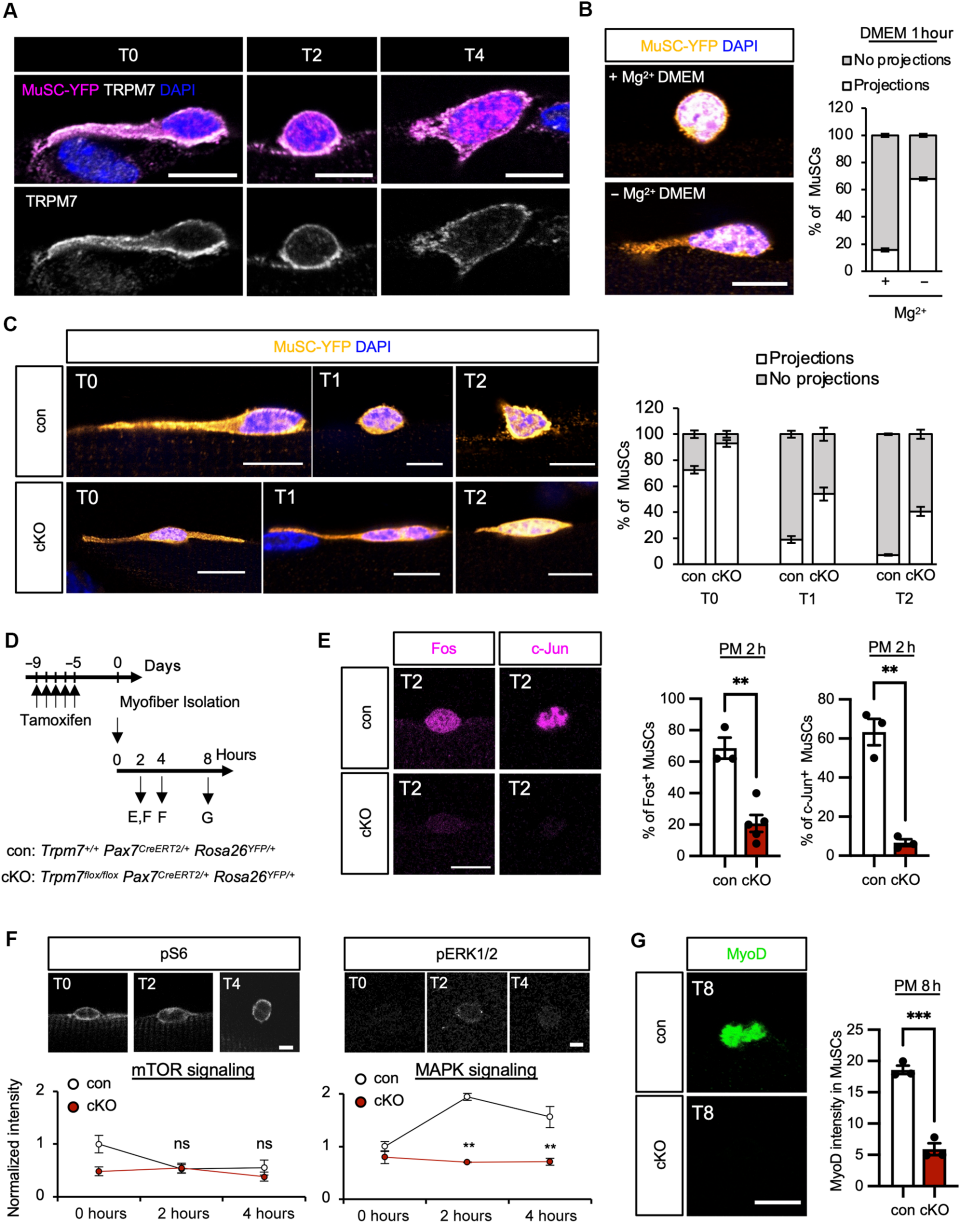
*Trpm7* deficiency impairs early responses required for MuSCs activation. (**A**) Evaluation of MuSCs with quiescent projection on myofibers from *Pax7^CreERT2/+^; Rosa26^YFP^*. TRPM7 expression (white) and YFP (magenta) were detected on myofibers freshly isolated (T0), or cultured for 1 hour (T1) and 2 hours (T2). MuSC morphology was visualized by detection of YFP (magenta). (**B**) MuSCs morphology cultured for 1 hour in DMEM with or without Mg^2+^. MuSC morphology was evaluated by detection of YFP (yellow). Nuclei were detected by DAPI (blue) (>50 MuSCs per condition from *N* = 3 mice). (**C**) MuSCs morphology in T0, T1, and T2 MuSCs. Left: Representative images of MuSCs. Top panels: con; bottom panels: cKO. YFP: yellow; nuclei (DAPI): blue. Right: Quantification of MuSCs with or without projections (>50 MuSCs per condition from *N* = 3 mice). (**D**) Time course for induction of *Trpm7*-deficiency, isolation, and culture of myofibers. (**E**) Expression of AP-1 transcription factor in T2 MuSCs. Left panels: Representative fluorescent images of AP-1 transcription factor (Fos and c-Jun). Quantification of AP-1 expression in T2 MuSCs (>50 MuSCs per condition from *N* = 3 mice). (**F**) Detection of pS6 (left) and phosphorylated ERK (pERK1/2; right) expression in T0, T2, and T4 MuSCs. Top: Representative images of con MuSCs. Bottom: Quantification of fluorescence intensity of pS6 and pERK1/2 normalized to con T0 MuSCs intensity. (**G**) MyoD expression in T8 MuSCs. Left: Representative fluorescent images of MyoD. Top: con; bottom: cKO. Right: Quantification of MyoD intensity (>50 MuSCs per condition from *N* = 3 mice). Scale bars: 10 μm in [(A), (B), (C), (E), (F), and (G)]. h, hours.

As TRPM7 regulates MuSC cell cycle transition, these data encouraged us to investigate whether TRPM7 functions in early activation of MuSCs. Kann *et al*. (*26*) reported a protocol for proper evaluation of MuSCs morphological phenotypes in isolated myofibers, which are equivalent to those in vivo (*26*). Leveraging this modified preparation, we found that TRPM7 is localized at the plasma membrane, while MuSCs have quiescent projections in freshly isolated (T0) MuSCs and early activated (T2-T4) MuSCs (Fig. 6A). This led us to investigate the function of Mg^2+^ and TRPM7 in regulating MuSC quiescent projections. Strikingly, Mg^2+^ depletion from the culture medium resulted in delayed retraction of quiescent projections (Fig. 6B). Also, approximately 90% of MuSC quiescent projections were retained in *Trpm7* cKO mice freshly isolated myofibers (T0), 60% after 1 hour of culture (T1), and 40% after 2 hours of culture (T2), time points at which most control MuSCs lose their projections (Fig. 6C). Furthermore, *Trpm7* gene deletion resulted in impaired induction of AP-1 proteins (Fos and c-Jun) in MuSCs at 2 hours post-myofiber culture (Fig. 6, D and E).

As TRPM7 is responsible for PI3K/Akt/mTORC1 pathway activation during the late activation response of cell cycle entry in MuSCs (Fig. 5C and fig. S6F), we examined pS6 as a downstream target of mTORC1 during the early phase of MuSCs activation. pS6 levels were unchanged in early activated control and *Trpm7* cKO MuSCs cultured for 2 or 4 hours (Fig. 6F). Also, Fos-positive MuSCs was unchanged by treatment with mTOR pathway inhibitors for 2 hours (fig. S7B). Previously reported inhibition of pathways (fig. S7A) such as MAPK (PD 0325901) and Rho-associated protein kinase (ROCK) (Y-27632) showed insufficient induction of Fos expression in MuSCs (fig. S7B). Inhibition of phospholipase C (U 73122), which was originally identified for binding to TRPM7 (*5*), failed to induce Fos-positive MuSCs (fig. S7B).

The MAPK pathway has been reported as the earliest signaling pathway for early activation of MuSCs (*25*). We evaluated phospho–extracellular signal–regulated kinase (ERK)1/2 (pERK) levels and found that pERK levels did not increase in *Trpm7* cKO MuSCs compared with control MuSCs cultured for 2 or 4 hours (Fig. 6F). In addition, MyoD induction was impaired in *Trpm7* cKO MuSCs on myofibers cultured for 8 hours (T8) (Fig. 6G). The up-regulation of Rho/ROCK signaling is also one of the earliest signaling pathways for the early activation of MuSCs (*26*). Consistently, *Trpm7* cKO MuSCs displayed a clear reduction in RhoA activity compared to controls (fig. S7C). Mg^2+^ depletion from the culture medium failed to induce of Fos (fig. S7D) and delayed up-regulation of MyoD (fig. S7E) and Ki67 (fig. S7F), strengthening the pivotal role of Mg^2+^ in MuSCs. Together, Mg^2+^ influx via TRPM7 is a primary event for regulating quiescent projections and activates the MAPK pathway but not mTOR pathway during early activation of MuSCs.

### Supplementation of Mg^2+^ restores *Trpm7* deficiency in MuSCs

**Fig. 7.**
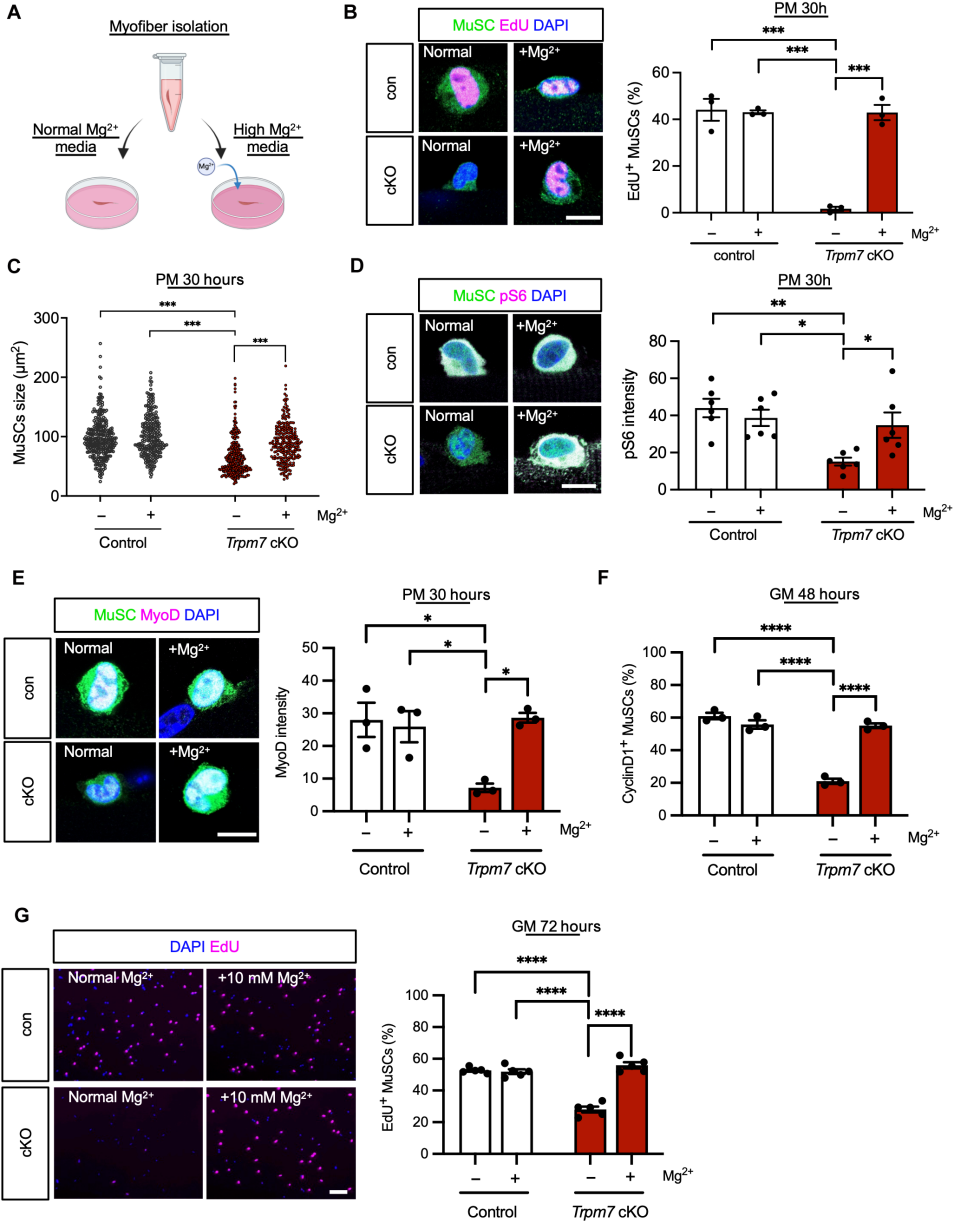
Mg^2+^ supplementation rescues defects of *Trpm7*-deficient MuSCs. (**A**) Experimental procedures for myofiber isolation and culture in plating medium (PM) supplemented with 10 mM Mg^2+^. (**B** to **E**) Evaluation of con and cKO MuSCs on myofibers cultured in PM supplemented with 10 mM Mg^2+^ for 30 hours. con or cKO mice harboring the *Rosa26-YFP* were used for these experiments. (B) EdU incorporation assay on YFP-positive MuSCs in con and cKO MuSC on myofibers. (>15 myofibers per group were investigated from *N* > 4 mice). (C) MuSCs cell size (>100 MuSCs per group were investigated from *N* = 3 mice). (D) pS6 expression in MuSCs on myofibers (>50 MuSCs per group were investigated from *N* = 6 mice). (E) MyoD expression in MuSCs on myofibers (>50 MuSCs per group were investigated from *N* = 3 mice). (**F**) Quantification of Cyclin D1–positive MuSCs of con and cKO MuSCs cultured in GM supplemented with 10 mM Mg^2+^ for 48 hours (>300 MuSCs per group were investigated from *N* = 3 mice). (**G**) EdU assay on MuSCs cultured in growth medium supplemented with 10 mM Mg^2+^ for 3 days. EdU was incorporated into MuSCs for 3 hours before fixation. Left panels: Representative images of EdU assays. Right: Quantification of EdU-positive MuSCs (>500 cells from *N* = 3 mice per condition). Scale bars, 10 μm in [(B), (D), and (E)] and 100 μm in (H). h, hours.

Last, as *Trpm7* deficiency lowers intracellular Mg^2+^ levels, we performed a rescue experiment by supplementation of extra MgCl_2_ to MuSCs (Fig. 7A). MuSCs in myofibers were cultured in medium supplemented with 10 mM MgCl_2_. The decreased number of EdU-positive cells observed in *Trpm7* cKO MuSCs was fully rescued by extra supplementation with 10 mM MgCl_2_ (Fig. 7B). The same trend was observed with cell size (Fig. 7C). To investigate the effect of Mg^2+^ supplementation on the downstream pathway, pS6 fluorescent intensity levels were measured (Fig. 7C). Mg^2+^ supplementation rescued the mTOR pathway, indicating that Mg^2+^ incorporation via TRPM7 activates the mTOR pathway in MuSCs (Fig. 7D). MyoD expression was partially rescued in an 8-hour short-term culture with Mg^2+^ supplementation (fig. S8A) and fully rescued by 30- or 72-hour long-term culture with Mg^2+^ supplementation (Fig. 7E and fig. S8B). Furthermore, *Trpm7* cKO MuSCs cultured for 48 hours in Mg^2+^ supplemented medium restored Cyclin D1–positive MuSCs ratio (Fig. 7F). Also, the proliferative defects observed in *Trpm7*-deficient MuSCs were abolished by culturing in medium supplemented with Mg^2+^ (Fig. 7G).

TRPM7 is known to be permeable to divalent cations including Ca^2+^ and Zn^2+^ (*43*), which are involved in a variety of cellular and physiological processes (*16*, *44*). To explore whether TRPM7 facilitates the transport of Ca^2+^ and Zn^2+^ in MuSCs during muscle regeneration, we used fluorescent indicators using Cal-520 and FluoZin-3, respectively. Cytosolic Ca^2+^ and Zn^2+^ elevated in activated MuSCs at 1 or 3 days after regeneration compared with uninjured MuSCs (fig. S9, A, B, E, and F), and both Ca^2+^ and Zn^2+^ levels returned to baseline levels by 7 to 28 days post-CTX injury (fig. S9, B and F). The increases in Ca^2+^ and Zn^2+^ levels were reduced in *Trpm7* cKO MuSCs (fig. S9, C, D, G, and H), confirming that TRPM7 mediates the influx of these ions in activated MuSCs. Notably, supplementation with either Ca^2+^ or Zn^2+^ failed to restore the proliferation defects observed in *Trpm7* cKO MuSCs, in clear contrast to Mg^2+^ supplementation (Fig. 7G and fig. S9I). Together, these results demonstrate that Mg^2+^ influx via TRPM7 is essential for MuSC cell cycle and proliferation.

## DISCUSSION

Tissue-resident stem cells allow our bodies to maintain homeostasis in response to external stimuli and injuries faced throughout life (*21*, *45*). However, it remains uncertain how MuSCs sense these injuries and initiate the regeneration processes. Here, we show that TRPM7, a nonselective cation channel activated by environmental changes, is functionally expressed in MuSCs. On the basis of our genetic engineering approach, we demonstrate that TRPM7 plays fundamental roles in muscle regeneration by promoting MuSCs to enter the cell cycle and proliferate in an extracellular Mg^2+^-dependent manner. Strikingly, *Trpm7* deficiency caused defects in the early responses of MuSCs including retraction of quiescent projections, immediate early gene expression, and induction of transition from G_0_ to G_Alert_. Thus, our results provide insights into the molecular mechanism by which initiation of MuSC activation and transition occurs during muscle regeneration processes.

Magnesium ion is known to be involved in a variety of cellular and physiological phenomena. In skeletal muscle, Mg^2+^ participates in regulation of muscle contraction by modulating the function of a Ca^2+^ pump in the sarcoplasmic reticulum (sarcoendoplasmic reticulum calcium transport adenosine triphosphatase or SERCA) (*46*). In addition, during muscle regeneration, Mg^2+^ levels stay elevated in MuSCs for at least 7 days (Fig. 1), when myofibers mature by myogenic cell fusion (*47*), indicating that Mg^2+^ influx participate in myoblast fusion as suggested in recent in vitro studies (*48*, *49*). Although these findings suggest the role of Mg^2+^ in muscle homeostasis and regeneration, the molecular entity that transduces Mg^2+^ to regulate these processes remains unidentified. Among a series of Mg^2+^-permeable ion channels and transporters, we identify TRPM7 as one of the main cation channels essential for muscle regeneration. Using the Cre/loxP system, we observed that increased Mg^2+^ was significantly reduced in *Trpm7*-deficient MuSCs (Fig. 1) and that muscle regeneration was impaired in *Trpm7*-deficient muscle after CTX injury (Fig. 2). Thus, we provide direct evidence that Mg^2+^ influx through TRPM7 is one of the earliest events for MuSCs to exit quiescence and enter the cell cycle, a process that is essential for proper muscle regeneration.

TRPM7 is also known as a “chanzyme” as it has an atypical kinase domain at the C terminus. Our data using kinase-inactive KR mice demonstrated that the TRPM7 kinase domain has no effect during muscle regeneration (fig. S3) and that the total number of proliferating MuSCs was comparable in KR mice and controls (fig. S5G). This supports our notion that the ion channel activity of TRPM7 is critical for MuSC proliferation. Intriguingly, the number of MuSCs entering the cell cycle in cultured myofibers at 30 hours (i.e. activated MuSCs) was significantly decreased in KR mice compared with controls (Fig. 4M), suggesting that TRPM7 kinase activity may be involved in MuSCs activation. Previous literature has shown that the kinase domain of TRPM7 is cleaved from the channel domain and translocates to the nucleus, whereupon it binds to chromatin remodeling complexes and phosphorylates specific serine/threonine residues in the histones of embryonic stem cells (*50*). Analyzing the epigenomic properties in MuSCs isolated from TRPM7-kinase mutant mice may provide a full understanding of the physiological significance of TRPM7.

Our RNA-seq analysis identified a series of TRPM7-dependent downstream processes in MuSCs including cell cycle progression, myogenesis, and mitochondrial function (Fig. 3). Intriguingly, we found that TRPM7 is required for MuSCs to transition from a G_0_ to G_Alert_ phase in response to CTX injury and in vivo administration of a recombinant HGFA (Fig. 5). Previous studies have shown that HGF and its receptor c-Met act as the upstream activator of the mTORC1 signaling pathway, which is essential for the G_0_-to-G_Alert_ transition in MuSCs (*42*). Here, we demonstrate that TRPM7 functions as an upstream membrane protein alongside c-Met. Harry Rubin proposed that Mg-ATP (rather than ATP) serves as the main substrate for phosphoryl transfer in the mTOR pathway (*51*). His MMM (membrane, magnesium, mitosis) model suggests that growth factor binding to membrane receptors induces a sustained increase in cytosolic Mg^2+^, which enhances protein synthesis and promotes proliferation. TRPM7 possibly contributes to this process by acting as the key reservoir of Mg^2+^ during the transition of MuSCs from the G_0_ to G_Alert_ phase.

Our findings with genetically modified mice demonstrate that TRPM7 is crucial for the early activation of MuSCs (Fig. 6). Early activation requires rapid translational activation for myogenic differentiation and protein synthesis, regulated by Eukaryotic initiation factor (eIF) phosphorylation and the mTOR pathway (*22*, *52*). While the molecular mechanisms initiating this early response are still under debate, our findings show that TRPM7 activates two key pathways—the ERK/MAPK pathway for early MuSCs activation and the mTOR pathway for G_Alert_ induction and S phase entry. Although the function of the mTOR pathway in MuSCs during muscle regeneration is well established (*53*, *54*), its involvement in the rapid response to injury remains unclear. Our pharmacological studies suggest that the ERK pathway, but not the mTOR pathway, drives MuSCs’ rapid activation (fig. S7B), while mTOR is fundamental for later stages such as S phase entry (fig. S6F). A recent study focused on the role of mTOR pathways during injury from the evolutionary perspective. Intriguingly, during acute injury, axolotl mTOR signaling is rapidly up-regulated by constitutive activation of mTOR protein, whereas mouse mTOR signaling is unchanged before and 2 hours after injury (*55*). Investigating how ion channels, such as axolotl TRPM7, regulate the mTOR pathway during tissue regeneration across various species is compelling.

We further found that TRPM7 is responsible for retraction of quiescent projections in an Mg^2+^-dependent manner (Fig. 6, A to C). Although a previous study reported that the Rac-to-Rho GTPase switch is a critical determinant for retraction of quiescent projections during MuSC activation (*26*), the upstream determinant is still elusive. Our results demonstrate that TRPM7 contributes to the activation of Rho (fig. S7C), suggesting that Mg^2+^ influx via TRPM7 may regulate the small GTPase family, which drives the retraction of neurite-like projections and is required for MuSC activation. In addition, TRPM7 has been previously shown to regulate cell migration by cytoskeletal rearrangement (*56*, *57*), further supporting its role in the regulation of Rho activity. A mechanosensitive ion channel, PIEZO1, is reported to be involved in generation of quiescent projections in vivo (*58*) and may be essential for sensing biophysical forces during muscle injury. As TRPM7 is reported to be a mechanosensitive ion channel (*17*), it is plausible that a series of mechanosensitive ion channels, including TRPM7 and PIEZO1, concertedly regulate quiescent projections during different MuSC states.

One limitation of our study is the inability to capture the earliest stages of Mg^2+^ dynamics during MuSC activation. While we used freshly isolated MuSCs loaded with the magnesium-sensitive dye MagGreen AM, this approach is constrained by the rapid changes in cellular characteristics that occur during and immediately after FACS isolation (*23*, *25*). These changes likely obscure the magnesium dynamics occurring within the first few hours of activation. A promising alternative is the use of genetically encoded magnesium indicators, such as Mag-FRET to enable in vivo imaging of MuSCs (*59*, *60*). This method would allow for precise temporal resolution of Mg^2+^ fluxes during the early phases of MuSC activation in their native environment.

Overall, we have identified a pathway for MuSC activation, in which magnesium ion influx via TRPM7 governs pathways to initiate MuSCs to exit from a quiescence to an active state. Our findings highlight an “Mg^2+^nificent” role of TRPM7 in MuSCs and provide insights into understanding of MuSC-dependent muscle regeneration and establishment of therapeutic options for muscle diseases.

## MATERIALS AND METHODS

### Mice

Animal care, ethical use, and protocols were approved by the Animal Care Use and Review Committee of the University of Shizuoka. All mice used in this study had a C57BL6 genetic background and were adults between 7 and 20 weeks old, with age matched controls. *Trpm7^+/+^*; *Pax7^CreERT2/+^* were used as control and *Trpm7^flox/flox^*; *Pax7^CreERT2/+^* were used as *Trpm7* cKO mice. Approximately equal numbers of male and female mice were used in all experiments. *Trpm7^flox/flox^* mice were provided from Y.M. at Kyoto University. *Trpm7^flox/flox^* mice were further mated with *Pax7^CreERT2/+^* transgenic mice (the Jackson Laboratory, strain ID:012476) (*61*) to generate MuSC-specific *Trpm7*-deficient mice. *TRPM7^KR^* mice were kindly provided by M.M. at University of the Ryukyus. The primers used for genotyping are listed in table S2. TMX (Sigma-Aldrich; T5648) dissolved in corn oil at a concentration of 20 mg/ml was used to induce Cre recombinase expression. The mice were injected intraperitoneally with 100 μl of TMX daily for five consecutive days.

### MuSCs isolation using FACS

MuSCs from the uninjured limb muscles were isolated as previously described (*62*). Briefly, skeletal muscle samples obtained from mouse limbs were subjected to collagenase treatment using 0.2% collagenase type I (Sigma-Aldrich). Mononuclear cells were incubated with phycoerythrin (PE)–conjugated anti-mouse Ly-6A/E (Sca-1) antibody (1:200; #122508, BioLegend), PE-conjugated anti-mouse CD45 antibody (1:200; #103106, BioLegend), PE-conjugated anti-mouse CD31 antibody (1:200; #102508, BioLegend), and allophycocyanin-conjugated anti-mouse CD106 antibody (1:100; #105718, BioLegend) at 4°C for at least 30 min. These cells were resuspended in PBS containing 2% fetal bovine serum (FBS) and then subjected to cell sorting to collect CD106-positive cells using MA900 (Sony) or BD FACSAria II.

### Reverse transcription polymerase chain reaction

For reverse transcription polymerase chain reaction (PCR) analysis, MuSCs were isolated from mice with FACS as described above. Total RNA was isolated using the QIAGEN RNeasy Micro Kit (#74104, QIAGEN). cDNA was generated using the PrimeScript II First strand cDNA synthesis kit (#6210A Takara). Quantitative PCR was performed with PowerUp SYBR Green Master Mix (Thermo Fisher Scientific) using the StepOne system (#A25741, Thermo Fisher Scientific). Copy numbers were determined using standard curves from the gene of interest and were compared to 18*S*. Relative expression was calculated using the 2^−ΔΔ*C*t^ method. The primers used are listed in table S2.

### Single myofiber isolation

Myofibers were isolated from the EDL muscles, as previously described (*62*). Isolated EDL muscle samples were incubated with 0.2% collagenase I (#C0130, Sigma-Aldrich) in Dulbecco’s modified Eagle’s medium (DMEM) at 37°C for 2 hours. Myofibers were released by gently flushing the muscle samples in plating medium using a fire-polished glass pipette. In Fig. 6, myofibers were prepared on the basis a modified protocol as described in (*26*). Isolated EDL muscle samples were incubated with 0.2% collagenase I (#C0130, Sigma-Aldrich) in DMEM at 37°C for 1 hour, and then myofibers were released by gently flushing the muscle samples in plating medium using a fire-polished glass pipette.

Custom Mg^2+^-free DMEM was purchased from Gmep Incorporated. For myofibers cultured in Mg^2+^-free DMEM, 0.2% collagenase I (#C0130, Sigma-Aldrich) was prepared using Mg^2+^-free DMEM.

### MuSC culture

MuSCs were cultured in growth medium [(DMEM; #043-30085 FUJIFILM Wako) supplemented with 30% fetal bovine serum (Sigma-Aldrich), 1% chicken embryo extract (#C3999 US Biological), basic fibroblast growth factor (10 ng/ml; #47079000, ORIENTAL YEAST Co., Ltd.), and 1% penicillin-streptomycin (#168-23191, FUJIFILM Wako)] on culture dishes coated with Matrigel (#354234, Corning). For MuSCs differentiation, the medium was changed to differentiation medium (DMEM supplemented with 5% horse serum and 1% penicillin-streptomycin) of MuSCs cultured for 6 days. Ten micromolar of EdU (#A10044, Invitrogen) was added to the growth medium during culture for EdU incorporation.

For MuSC growth on floating myofibers, isolated myofibers were cultured in plating medium [DMEM (#043-30085, FUJIFILM Wako) supplemented with 10% horse serum (Sigma-Aldrich), 0.5% chicken embryo extract (#C3999, US Biological), and 1% penicillin-streptomycin (#168-23191, FUJIFILM Wako)] at 37°C with 5% CO_2_. Five micromolar EdU (#A10044 Invitrogen) was added to the plating medium for incorporation to analyze S phase entry.

For rescue experiments, isolated MuSCs or myofibers were cultured in growth medium or plating medium supplemented with 10 mM MgCl_2_ or NaCl. Myofibers were treated with mTOR inhibitors rapamycin (100 nM; #553211, Sigma), INK128 (500 nM; #CS-0557, ChemScene LLC), 4EGI-1 (50 μM; #15362, Cayman Chemical, MAPK kinase inhibitor PD98059 (50 μM; # 72172, STEMCELL Technologies), PLC inhibitor U73122 (10 μM; #S8011, Selleck Chemicals), and ROCK inhibitor Y-27632 (10 μM; #72302, STEMCELL Technologies). MuSCs were treated with mTOR inhibitors rapamycin (100 nM; #553211, Sigma-Aldrich), INK128 (500 nM; #CS-0557, ChemScene LLC), and 4EGI-1 (50 μM; #15362, Cayman Chemical) in growth medium.

### Magnesium imaging (MagGreen and Mag-Fura-2)

Intracellular magnesium levels were detected by minor changes in protocols introduced by previous studies (*33*, *63*). Briefly, MuSCs were plated on glass-bottomed dish (#D11531H, Matsunami) and loaded with 2 μM Mag-Fura-2–AM (#M1292, Invitrogen) in Mg^2+^-free Margo’s solution (45 mM NaCl, 90 mM KCl, 2.5 mM CaCl_2_, 20 mM Hepes, and 10 mM glucose) and 2 μM Pluronic F-127 (#P2443-250G, Sigma-Aldrich) for 30 min at 37°C (5% CO_2_). Cells were washed and imaged in Mg^2+^-free Margo’s solution within a temperature controlled environmental chamber (set at 37°C) using a Zeiss live cell imaging system. After 60 s of baseline recording 10 mM MgCl_2_ was given. Images were obtained every 5 s using a 20× objective and quantified.

For in vivo cytosolic Mg^2+^ detection, freshly isolated MuSCs were directly sorted into 1.5-ml tubes containing growth medium. Then, 2 μM magnesium indicator Magnesium Green, AM (#M3735, Invitrogen), and 2 μM Pluronic F-127 (#P2443-250G, Sigma-Aldrich) were added into the sorted tubes before incubating for 30 min at 37°C (5% CO_2_). The stained MuSCs were sorted with SONY MA900 for analysis.

### Calcium imaging (Cal-520)

In vivo cytosolic Ca^2+^ was detected by fluorogenic calcium-sensitive dye Cal-520 (#21130, AAT Bioquest). Freshly isolated MuSCs were directly sorted into 1.5-ml tubes containing growth medium. Then, 10 μM calcium indicator Cal-520, AM, and 2 μM Pluronic F-127 (#P2443-250G, Sigma-Aldrich) were added into the sorted tubes before incubating for 30 min at 37°C (5% CO_2_). The stained MuSCs were sorted with SONY MA900 and detected with fluorescein isothiocyanate (FITC) for analysis.

### Zinc imaging (FluoZin-3)

In vivo cytosolic Zn^2+^ was detected by Zn^2+^-selective indicator FluoZin-3 (#F24194 Thermo Fisher Scientific). Freshly isolated MuSCs were directly sorted into 1.5-ml tubes containing growth medium. Then, 2 μM Zn^2+^-selective indicator FluoZin-3, AM, and 2 μM Pluronic F-127 (#P2443-250G, Sigma-Aldrich) were added into the sorted tubes before incubating for 30 min at 37°C (5% CO_2_). The stained MuSCs were sorted with SONY MA900 and detected with FITC for analysis.

### Mitochondria and RNA measurement using FACS

MuSCs were isolated from control (*Trpm7^+/+^; Pax7^CreERT2/+^; Rosa26^YFP^*) and *Trpm7* cKO (*Trpm7^flox/flox^; Pax7^CreERT2/+^; Rosa26^YFP^*) mice. PyroninY (40 nM; #sc-203755, Santa Cruz Biotechnology, for RNA content measurements), and MitoTracker Deep Red (40 nM; #M46753, Thermo Fisher Scientific, for mitochondrial content measurements) were added to collagenase-treated muscle samples. After incubation for 30 min at 37°C, YFP-positive cells were analyzed for gating the satellite cell population.

### 3′ untranslated region (3′UTR) RNA-seq

MuSCs were isolated from control (*Trpm7^+/+^; Pax7^CreERT2/+^; Rosa26^YFP^*) and *Trpm7* cKO (*Trpm7^flox/flox^; Pax7^CreERT2/+^; Rosa26^YFP^*) mice. Three thousand cells were sorted into 40 μl of 1× direct lysis buffer (TaKaRa, #635013) with ribonuclease inhibitor (TaKaRa, #2313A), and lysate was used for reverse transcription. Library preparation was performed using the CEL-Seq2 protocol (*64*) with a modification that Second Strand Synthesis Module (NEB) was used for double-stranded cDNA synthesis. Library was amplified by PCR without any sample pooling. Sequencing was carried out on Illumina NovaSeq6000, and the following quantitative analysis was performed using 81 base pair (bp) of insert reads (Read2).

### Data processing (3′UTR RNA-seq)

Cell barcode and UMI in Read1 was extracted by using UMI-tools (version 1.1.4) with following command “umi_tools extract -I read1.fastq --read2-in=read2.fastq –bc pattern=NNNNNNNNNNCCCCCCCCC”. Adaptor sequence and low-quality sequence were removed, read length below 20 bp was discarded by using Trim Galore (version 0.6.10), and reads were mapped to the GRCm38 reference using HISAT2 (version 2.2.1). Read counts for each gene were obtained by featureCounts (version 2.0.6) and UMI duplications were removed by UMI-tools. Four cell barcodes “ATGCAATGC, GACACGACA, CGATGCGAT, and TCTGGTCTG” were assigned to each sample and differentially expressed genes were extracted by DESeq2 (ver.1.30.1) using |log2FC| > 1.5 and *P* adj < 0.05 as threshold values.

### Immunofluorescent analysis

The cells were placed in Matrigel-coated glass-bottomed dishes and fixed with 4% paraformaldehyde (PFA)/PBS for 10 min. After permeabilization in 0.5% Triton X-100/PBS for 10 min, the samples were blocked in 1% bovine serum albumin (BSA)/PBS for 1 hour and probed with the antibodies listed in table S1 at 4°C overnight. After multiple washes with PBS, the secondary antibodies were added as listed in the table S1. Nuclei were detected using 4′,6-diamidino-2-phenylindole (DAPI) (1:1000; #D523 Dojindo).

Myofibers were fixed with 2% PFA/PBS for 5 min. After permeabilization and blocking with 0.5% Triton X-100 in 1% BSA/PBS for 15 min, the samples were probed with the antibodies listed in the table S1 at 4°C overnight. After washing once with PBS, the secondary antibodies listed in the table S1 were added. Nuclei were detected using DAPI (1:1000; #D523 Dojindo). Immunofluorescent signals were visualized with Alexa Fluor 488– or Alexa Fluor 555–conjugated secondary antibodies using an epifluorescence microscope (Axio Observer.Z1, Zeiss) or a confocal microscope (LSM 800, Zeiss) with a 63× objective lens. The fluorescence intensity was quantified using ImageJ software for statistical analyses. MuSC size was determined by YFP area from control (*Trpm7^+/+^; Pax7^CreERT2/+^; Rosa26^YFP^*) and *Trpm7* cKO (*Trpm7^flox/flox^; Pax7^CreERT2/+^; Rosa26^YFP^*) mice by using ImageJ. For EdU detection, a click reaction was performed after primary and secondary staining using a Click-iT EdU Imaging Kit (#C10337 and #C10338, Invitrogen) according to the manufacturer’s instructions.

### In situ binding assay for GTPase activity

Rho-GTPase activity in MuSCs on freshly isolated myofibers was measured by modified protocol in (*62*). Briefly, freshly isolated myofibers were immediately fixed with 4% PFA for 10 min. The MuSCs were then washed with PBS and permeabilized with 0.2% Triton X-100/PBS for 10 min. Myofibers were then incubated for 1 hour with glutathione *S*-transferase (GST)–tagged Rhotekin-Rho binding domain (Cytoskeleton RT01) proteins (50 μg/ml) diluted in 5% FBS/PBS. After washing with PBS twice, cells were incubated in anti-GST Alexa Fluor 488 (1:500 dilution; Invitrogen).

### Histological analysis

Cardiotoxin experiments were performed as previously described (*62*). Fifty microliter of 10 μM CTX (#L8102, Latoxan) was injected into the TA muscle of 8- to 15-week-old mice. The muscle samples were harvested at the time points indicated in each figure and snap-frozen in isopentane cooled with liquid nitrogen. Cross-cryosections (thickness: 7 μm) of the muscle samples were used for hematoxylin and eosin staining, as previously described (*62*). Cross-section area and fluorescence intensity were quantified using the ImageJ software for statistical analyses. For in vivo EdU uptake assay, EdU was dissolved in PBS at 0.5 mg/ml and injected intraperitoneally at 0.1 mg per 20 g body weight at the time points indicated in each figure.

### In vivo HGFA injection

Recombinant HGFA (#1200-SE, R&D Systems) was administered to mice at a dose of 1 μg diluted in 200 μl of PBS via tail vein injection as previously described (*42*). Control injections were administered by tail vein injection using 200 μl of PBS. The EDL muscle was isolated two days after HGFA injection to assess MuSCs on EDL myofibers.

### Data mining

Available RNA-seq datasets were retrieved from the public Gene Expression Omnibus (GEO) repository (www.ncbi.nlm.nih.gov/geo) website under accession number GSE104389 (*65*).

### Statistical analysis

Statistical analyses were performed using Microsoft Excel, PRISM, or JMP 11 (JMP Statistical Discovery LLC). The statistical significance of the differences between the mean values was analyzed using a nonpaired *t* test (two sided). Multiple comparisons were performed using Tukey’s test followed by analysis of variance (ANOVA). *P* values of *P < 0.05, **P < 0.01, ***P < 0.001, and ****P < 0.0001 were considered statistically significant. The results are presented as the means ± SEM. ns indicates results that are not statistically significant.
