# Supplementary material for: Mg2+ influx mediated by TRPM7 triggers the initiation of muscle stem cell activation
Source: Sci Adv. 2025 Apr 4;11(14):eadu0601. doi: 10.1126/sciadv.adu0601 (PMC11970462; doi:10.1126/sciadv.adu0601)
Supplement: Supplementary file 1 — Figs. S1 to S9 Tables S1 and S2 [file sciadv.adu0601_sm.pdf]

Science Advances
AAAS

Supplementary Materials for

Mg^{2+} influx mediated by TRPM7 triggers the initiation of muscle stem cell activation

Kotaro Hirano *et al.*

Corresponding author: Kotaro Hirano, hirano_k@u-shizuoka-ken.ac.jp; Yuji Hara, yhara@u-shizuoka-ken.ac.jp

Sci. Adv. **11**, eadu0601 (2025)
DOI: 10.1126/sciadv.adu0601

This PDF file includes:

Figs. S1 to S9
Tables S1 and S2

Supplementary Figure. 1

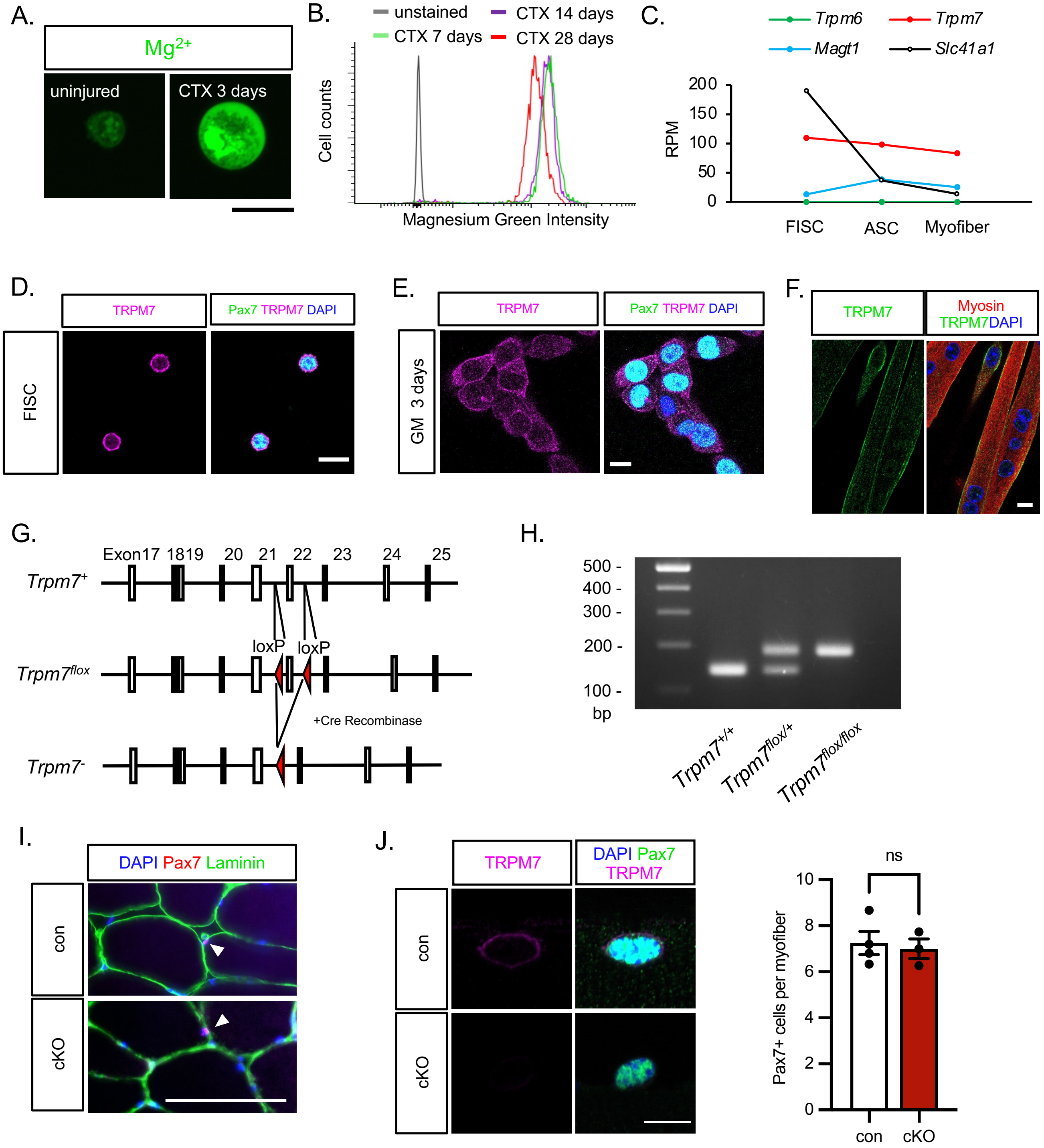

Supplementary Figure 1. Expression of Mg^{2+} permeable ion channel TRPM7 in MuSCs.

(**A** and **B**) Mg^{2+} measurements in MuSCs using Magnesium Green™, AM. (**A**) Image of MuSCs isolated from uninjured and three days after injury of skeletal muscle. (**B**) Cytosolic Mg^{2+} measurements using Magnesium Green™, AM in MuSCs isolated from skeletal muscle (Freshly isolated: uninjured 0- day, or after cardiotoxin injection 7-, 14-, 28- days) and analysis by flow cytometry. Grey, green, purple and red histogram indicates unstained MuSCs and MuSCs isolated from muscle injured for 7-, 14-, 28- days respectively.

(**C**) In silico analysis of magnesium transporters expressed in MuSCs. Target genes were quantified by reads per million (RPM) in freshly isolated MuSCs (FISC), activated MuSCs (ASC), and myofibers.

(**D** – **F**) Detection of TRPM7 protein in freshly isolated MuSCs. Immunofluorescent analysis of Pax7 and TRPM7 on freshly isolated MuSCs (**D**) or MuSCs cultured for 3 days (**E**). Detection of TRPM7 protein in differentiated myotubes (**F**).

(**G**) Schematic diagram representing the *Trpm7* locus in cKO mice.

(**H**) A representative agarose gel electrophoresis image of PCR products.

(**I**) Detection of MuSCs on muscle section. Representative immunohistochemical images of TA muscle sections, 1 month following the first TMX injection.

(**J**) Detection of MuSCs on isolated myofibers. Representative images of MuSCs on myofibers. TRPM7 antibody was used to confirm TRPM7 knockout. The number of Pax7+ cells per myofiber were quantified. (> 15 myofibers per condition from N > 3 mice).

Scale bars: 10 μm in (A), (D), (E), (F) and (J) and 100 μm in (I).

Supplementary Figure. 2

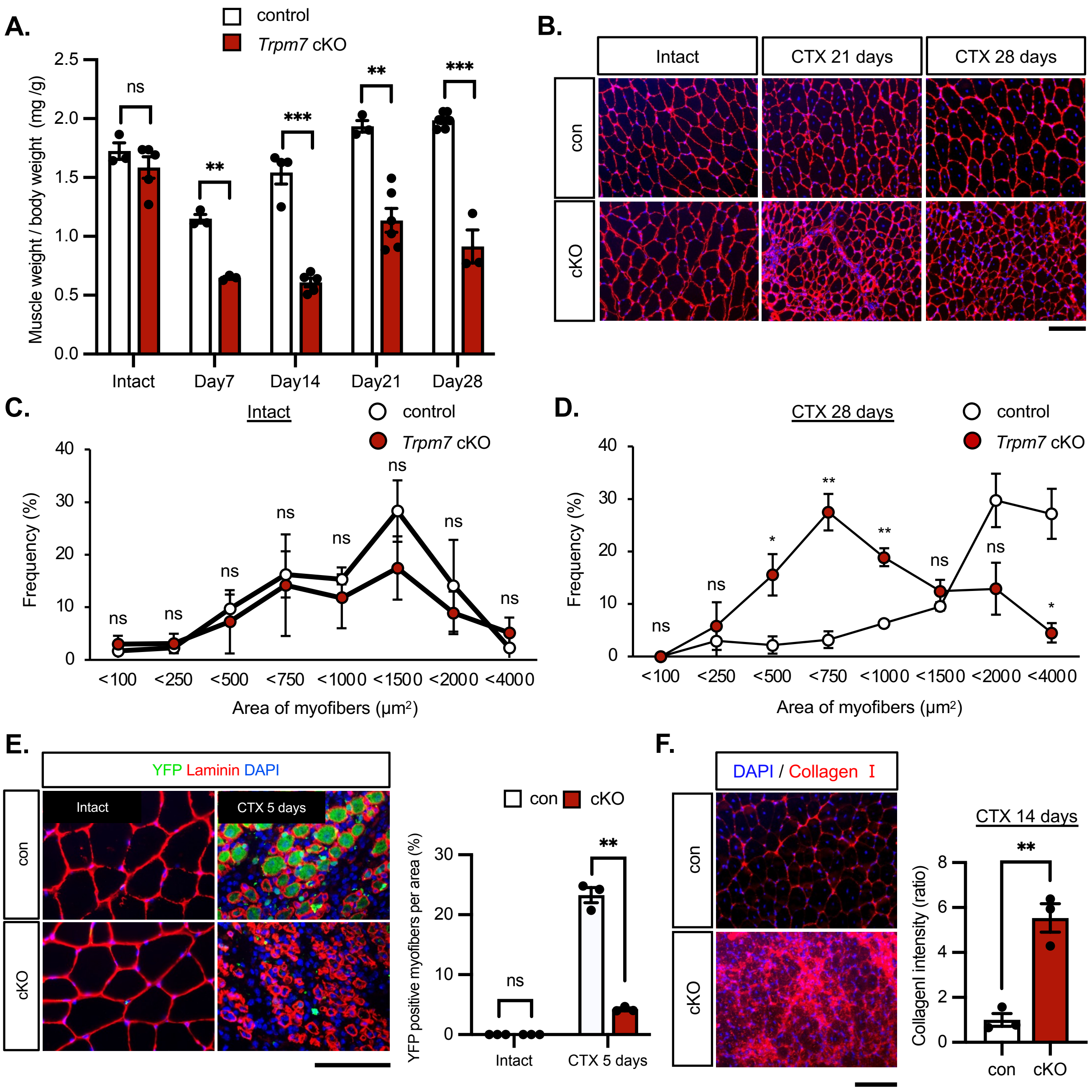

Supplementary Figure 2. TRPM7 is essential for muscle regeneration after skeletal muscle injury.

(**A**) Quantification of the ratio of muscle weight (mg) per mouse body weight (g) of TA muscle samples after CTX-induced muscle injury (N = 3 – 6 mice per condition).

(**B**) Representative images of immunohistochemistry staining of intact and regenerating TA muscle samples in con (upper panels) and cKO mice (lower panels). Red: Laminin I; blue: nuclei (DAPI) (N > 3 mice per condition).

(**C** and **D**) Quantification of CSA in B, intact (**C**) and 28 days post injured muscle (**D**). The percentage of CSA in total fibers were shown as the histogram.

(**E**) Representative images of intact and injured (4 days) TA muscle sections in YFP mice (upper panel) and cKO YFP mice (lower panel). Quantification of YFP+ area. (N = 3 mice per condition).

(**F**) Fluorescent intensity of collagen I in muscle sections 14 days post-cardiotoxin injection. The y axis shows the mean collagen I fluorescence intensity (ratio). Red: collagen I; blue: nuclei (DAPI). Scale bar: 100 μm in (B), (E), (F).

Supplementary Figure. 3

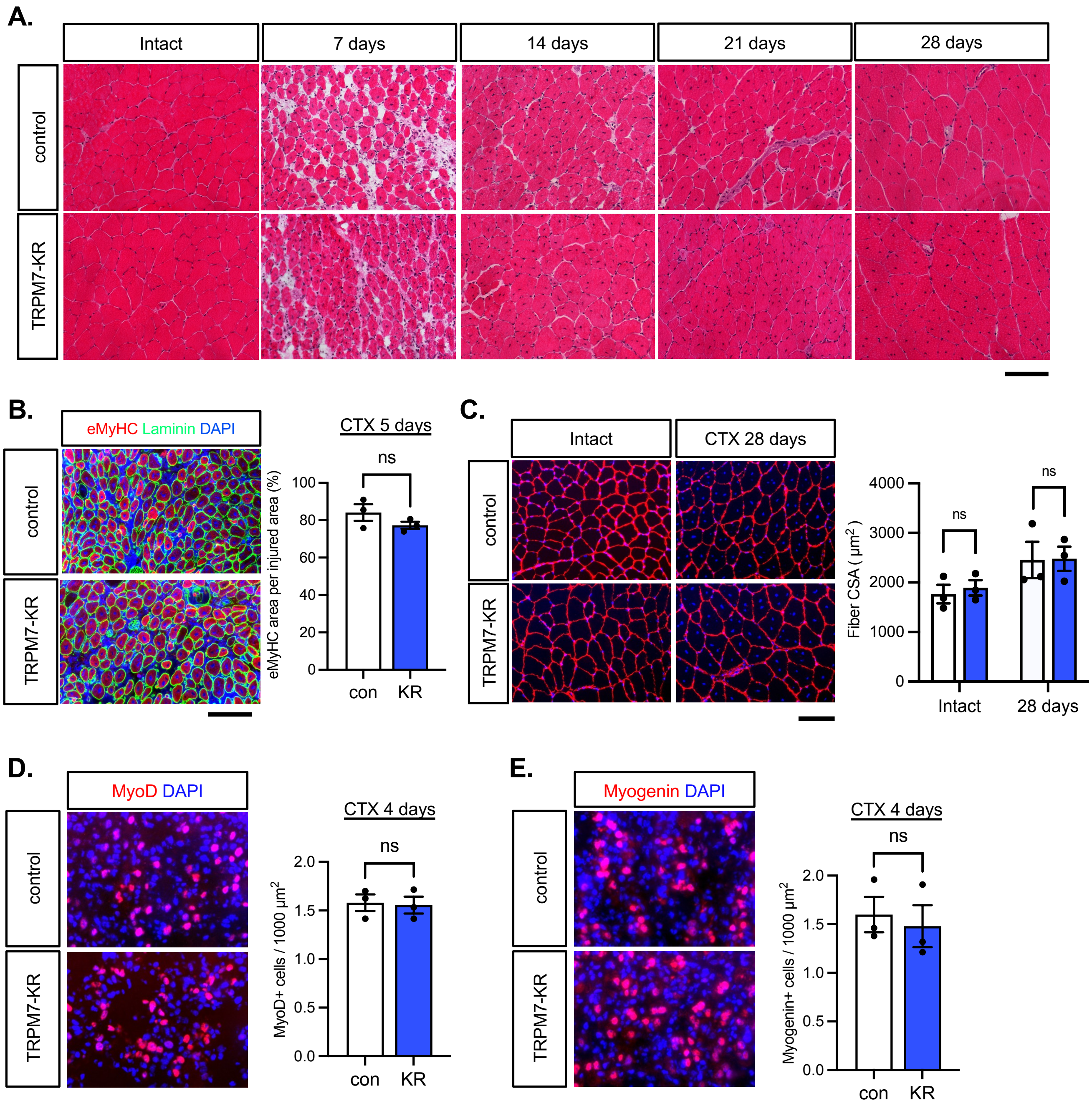

Supplementary Figure 3. TRPM7 kinase domain does not contribute to muscle regeneration after skeletal muscle injury.

(**A**) Hematoxylin and eosin staining of cross sections from intact and CTX-injected TA muscle samples harvested at indicated time points. Upper panels: control; lower panels: TRPM7-KR mice.

(**B**) Detection of embryonic myosin heavy chain (eMyHC) in cross-sections from control (upper panel) and TRPM7-KR muscle (lower panel). The area of eMyHC per section was evaluated 5 days after CTX treatment (N = 3 mice per condition).

(**C**) Quantification of CSA in intact and 28 days post injured muscle. (N = 3 mice per condition).

(**D**) Detection of MyoD-positive cells in cross-sections from control (upper panel) and TRPM7-KR muscle (lower panel). MyoD-positive cells per 1000 μm^2 were evaluated 4 days after CTX treatment. (N = 3 mice per condition).

(**E**) Detection of Myogenin-positive cells in cross-sections from control (upper panel) and TRPM7-KR muscle (lower panel). Myogenin -positive cells per 1000 μm^2 were evaluated 4 days after CTX treatment. (N = 3 mice per condition).

Scale bar: 100 μm in (B), (C), (D), (E).

Supplementary Figure. 4

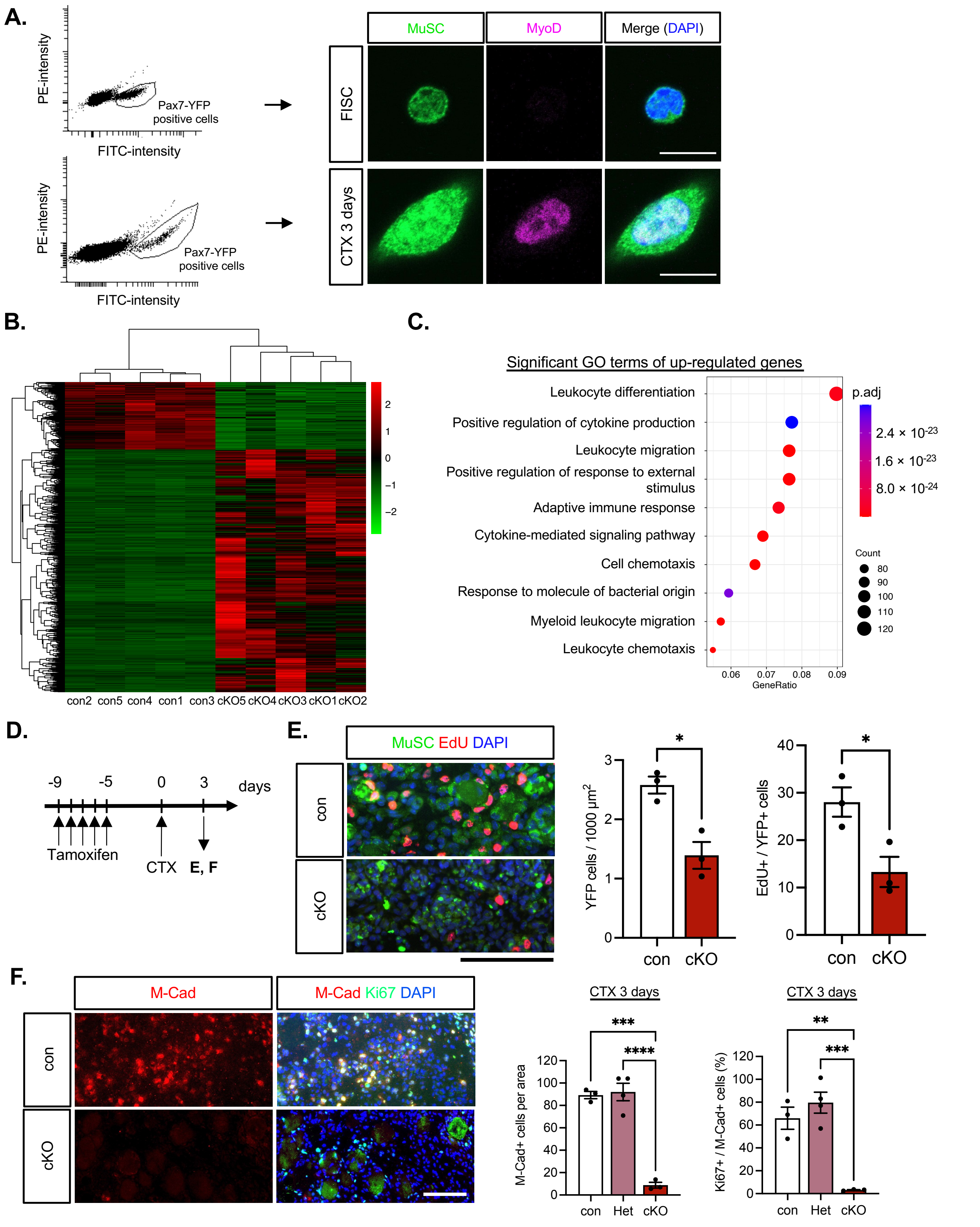

Supplementary Figure 4. *Trpm7*-deficient MuSCs show proliferative defect during muscle regeneration.

(**A**) Isolation of freshly isolated MuSCs and activated MuSCs. FACS profile shown to isolated YFP-positive MuSCs from mice. Isolated freshly isolated MuSCs and activated MuSCs were confirmed by the detection of MuSC activation marker MyoD.

(**B**) Heatmap showing the differentially expressed genes from RNA-seq.

(**C**) Gene ontology (GO) enrichment analysis of significantly up regulated genes in cKO.

(**D**) Time course for the induction of *Trpm7* deficiency, injection of TA with cardiotoxin, and isolation of regenerating muscle samples.

(**E**) EdU incorporation assay for regenerating TA muscle. After cardiotoxin administration, the number of EdU+ YFP+ cells (i.e., MuSCs possessing proliferative capacity) were counted in cross-sections from con and cKO muscle samples (N = 3 mice per conditions). Representative images of EdU (Red)- and YFP (green)-positive MuSCs in the con (upper panel) and cKO (lower panel) sections. The number of YFP-positive cells per 1000 μm^2 and the ratio of EdU-positive per YFP-positive cells were quantified. (N = 3 mice per conditions).

(**F**) Detection of Ki67 in MuSCs in regenerating TA muscle. After cardiotoxin administration, the number of Ki67+ M-Cadherin+ cells (i.e., MuSCs possessing proliferative capacity) was counted in cross-sections from con, hetero KO, cKO muscle samples. (N > 3 mice per condition). Representative images of Ki67 (green)- and M-cadherin (red)-positive MuSCs in the con (upper panel) and cKO (lower panel) sections. Number of M-cadherin+ cells per area and the ratio of Ki67-positive per M-cadherin-positive cells were quantified. (N = 3 mice per conditions).

Scale bars: 10 μm in (A) and 100 μm in (E) and (F).

Supplementary Figure. 5

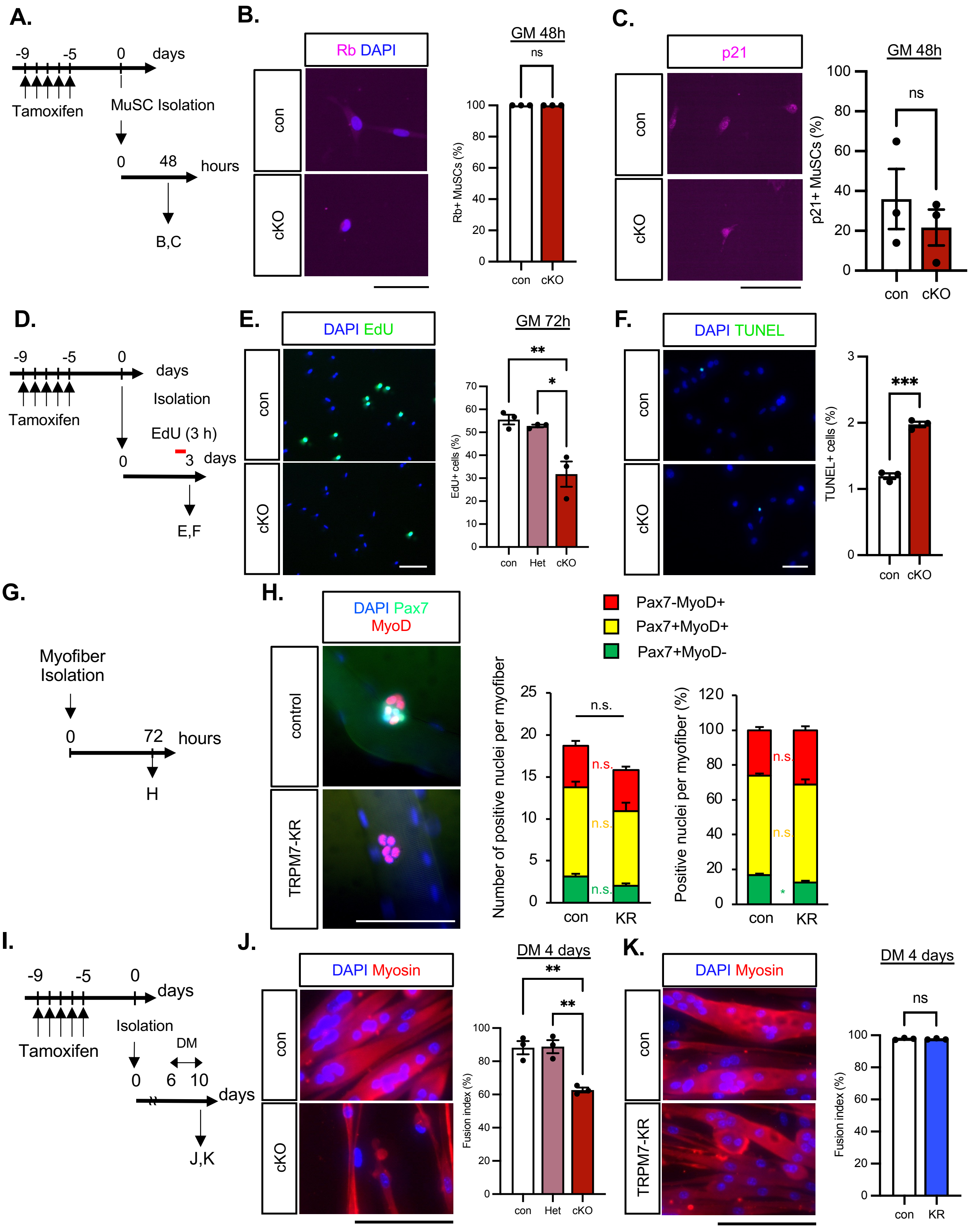

Supplementary Figure 5. *Trpm7*-deficiency reduces cell cycle entry and proliferative ability of MuSCs

(**A**) Time course for induction of *Trpm7*-deficiency and isolating and culturing MuSCs.

B and **C** Detection of Total Rb-positive and p21-positive MuSCs. Representative images of Total Rb (**B**) or p21 (magenta) (**C**) respectively. Quantification of the ratio of MuSCs. (> 300 MuSCs per condition from N = 3 mice).

D – **E** (**D**) Time course for induction of *Trpm7*-deficiency and isolating and culturing MuSCs. (**E**) EdU incorporation assay in cultured MuSCs. EdU was incorporated into MuSCs for 3 h prior to fixation (> 500 cells from N = 4 mice per condition). (**F**) TUNEL assay in cultured MuSCs. (> 300 MuSCs per condition from N = 3 mice).

G and **H** (**G**) Time course for harvesting and culturing of myofibers from TRPM7-KR mice. (**H**) Immunofluorescent analysis of MuSCs on floating myofibers cultured for 72 hours. Representative images of Pax7 (green), MyoD (red), and nuclei (blue) on control (upper panel) or TRPM7-KR (lower panel) myofibers. Evaluation of Pax7 and MyoD expression in control and TRPM7-KR. (> 15 myofibers per condition from N = 5 mice).

I – **K** (**I**) Time course for induction of *Trpm7*-deficiency and isolating, culturing and differentiating MuSCs. Images of differentiated myotubes. The ratio of nuclei inside myosin positive cells was quantified as fusion index. (N = 3 mice) (**J** and **K**).

Scale bars: 50 μm in (B) and 100 μm in (C), (E), (G), (I) and (J).

Supplementary Figure. 6

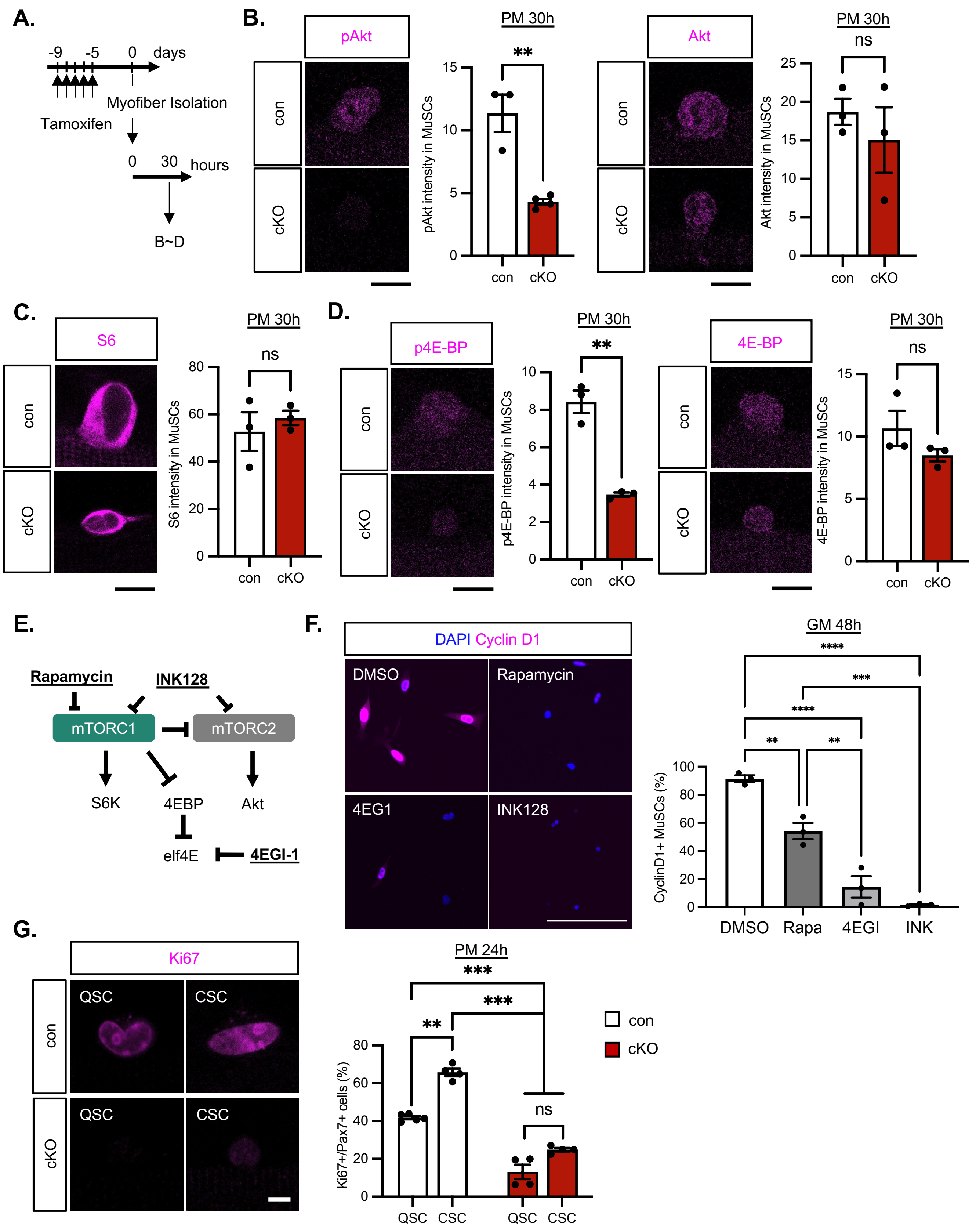

Supplementary Figure 6. The TRPM7-mTORC signalling axis controls cell cycle progression
(**A**) Time course for induction of *Trpm7*-deficiency and isolation of myofibers.
B – **D** Detection of downstream pathways of mTORC1 in MuSCs on myofibers cultured for 30h. (**B**) Detection of phosphorylated Akt (pAkt) and Total Akt in MuSCs. Fluorescent intensity detected and quantified. (>50 cells per condition from N = 3). (**C**) Detection of Total S6 in MuSCs. Fluorescent intensity detected and quantified. (>50 cells per condition from N = 3). (**D**) Detection of phosphorylated 4E-BP (p4E-BP) and Total 4E-BP in MuSCs. Fluorescent intensity detected and quantified. (>50 cells per condition from N = 3).
(**E**) Schematic demonstrating pathways of the mTOR pathway. Rapamycin (Rapa) inhibits mTORC1. 4EGI inhibits elf4E. INK128 (INK) inhibits mTORC1 and mTORC2.
(**F**) Pharmacological inhibition of the mTOR pathway restricts cell cycle progression. Cell cycle progression was quantified by detecting CyclinD1 in MuSCs cultured for 48 hours. DMSO was used for control. (>300 cells per condition from N = 3 mice).
(**G**) Detection of Ki67 in QSC and CSC on myofibers from control and *Trpm7* cKO mice cultured for 24 h in plating medium. (> 10 myofibers per condition from N = 4 mice).
Scale bars: 10 µm in (B), (C), (D) and (G), and 100 µm in (F).

Supplementary Figure. 7

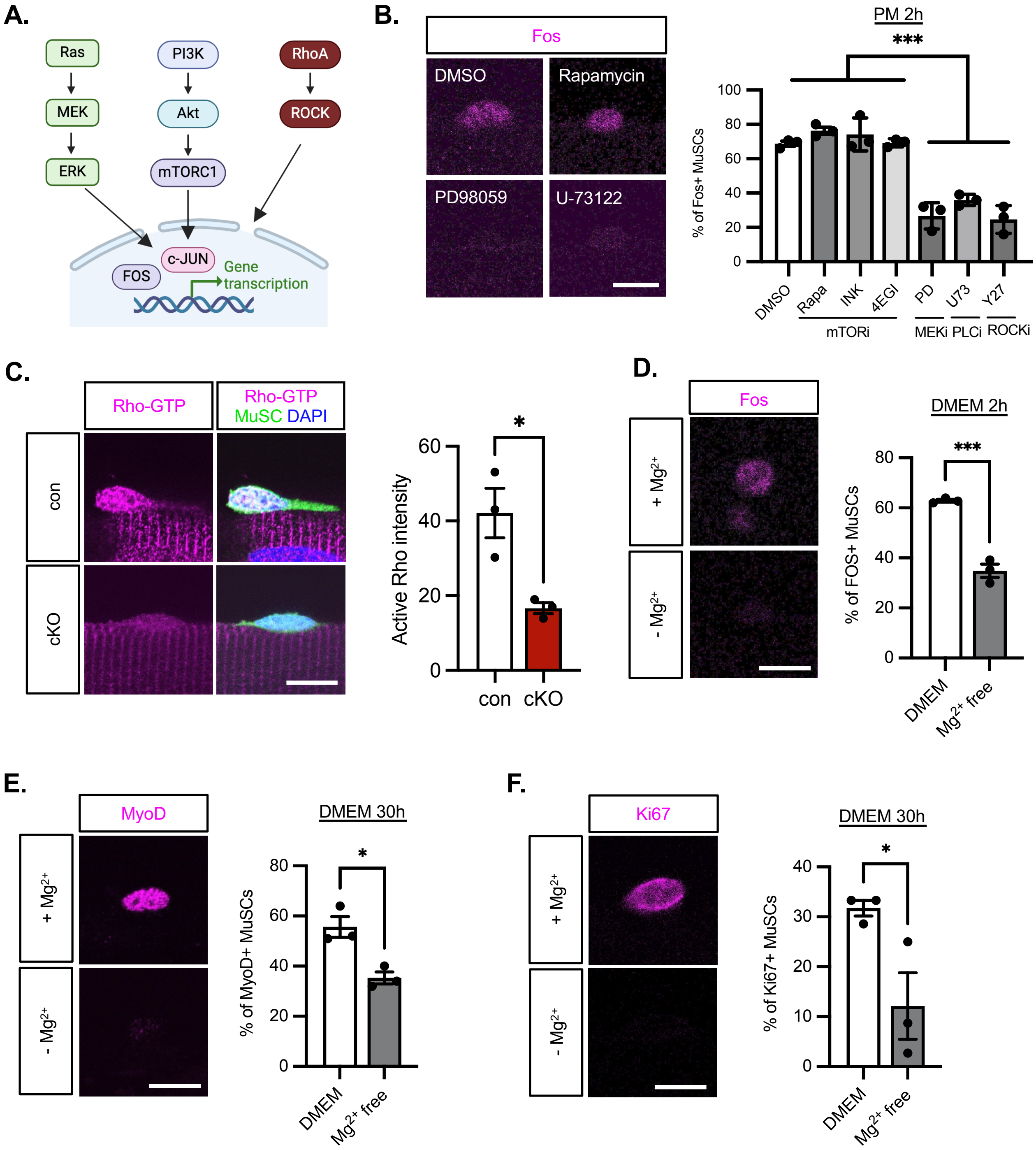

Supplementary Figure 7. MuSCs early activation is dependent on Mg^{2+} and the ERK pathway but not on mTORC pathway

(**A**) Schematic demonstrating expected pathways for Fos induction.

(**B**) Pharmacological inhibition of pathways during MuSCs early activation. (>50 cells per condition from N = 3).

(**C**) Image and quantification of Rho activity in control and cKO MuSCs in freshly isolated myofibers. (> 30 MuSCs per condition from N = 3 mice).

(**D**) Image and quantification of Fos expression in MuSCs cultured in DMEM in DMEM $\pm Mg^{2+}$ for 2 hours. (>50 cells per condition from N = 3).

(**E**) Image and quantification of MyoD expression in MuSCs cultured in DMEM $\pm Mg^{2+}$ for 30 hours. (> 15 myofibers per condition from N = 3 mice).

(**F**) Image and quantification of Ki67 expression in MuSCs cultured in DMEM $\pm Mg^{2+}$ for 30 hours. (> 15 myofibers per condition from N = 3 mice).

Scale bars: 10 µm in (B), (C), and (D).

Supplementary Figure. 8

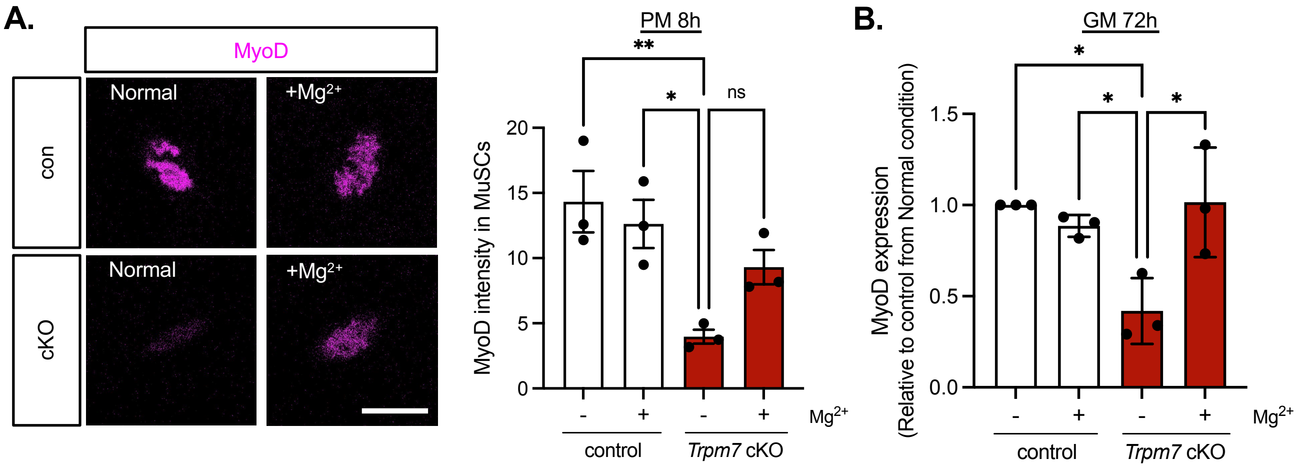

Supplementary Figure 8. Long term Mg^{2+} supplementation rescues MyoD expression of *Trpm7*-deficient MuSCs.

(**A**) Evaluation of con and cKO MuSCs on myofibers cultured in PM supplemented with 10 mM Mg^{2+} for 8h. Con or cKO mice harbouring the *Rosa26-YFP* were used for these experiments. MyoD expression in MuSCs on myofibers. (> 50 MuSCs per group were investigated from N = 3 mice).

(**B**) qPCR analysis of MuSCs cultured in growth medium supplemented with 10 mM Mg^{2+} for 72 h.

Scale bar: 10 μm

Supplementary Figure. 9

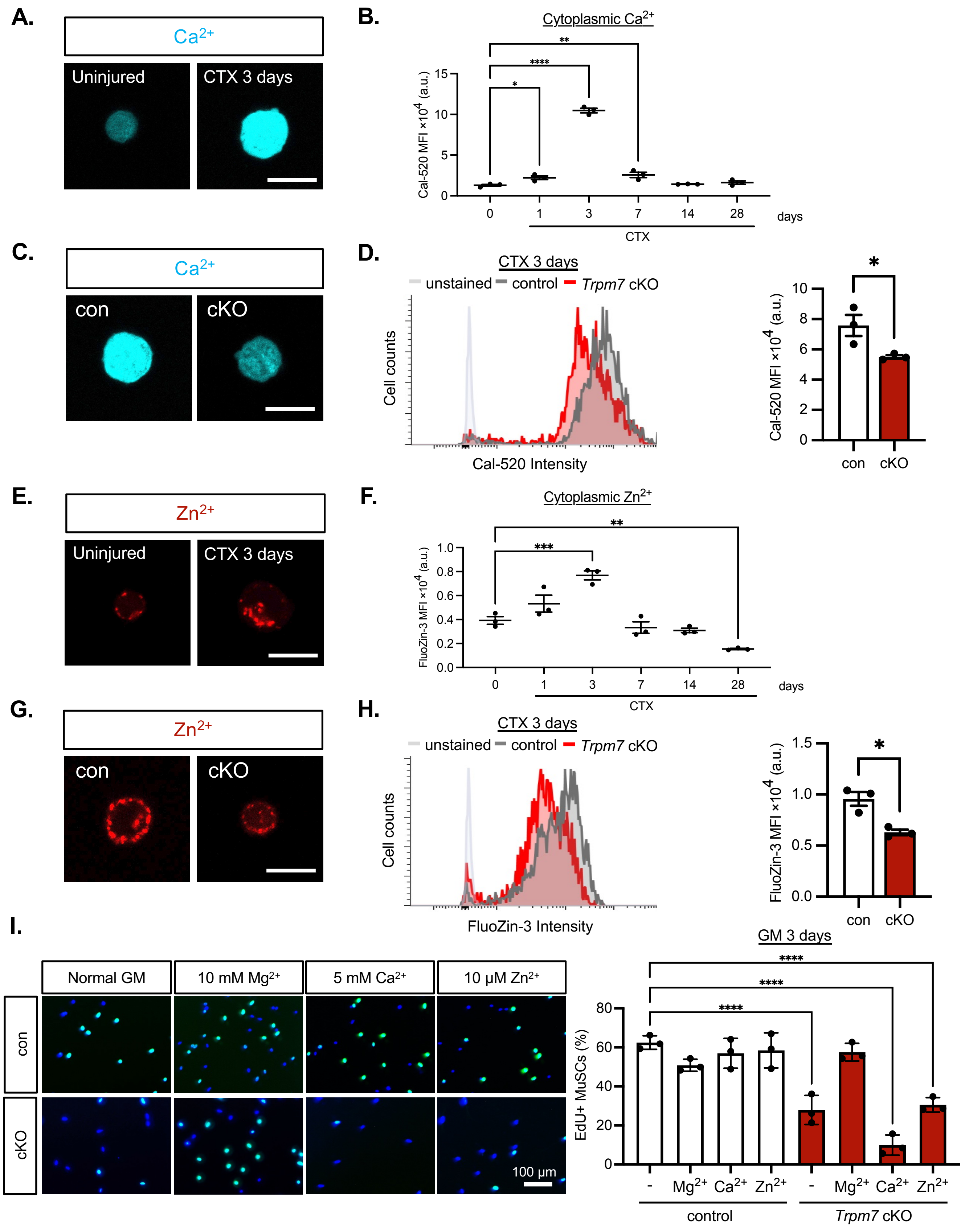

Supplementary Figure 9. TRPM7 mediates Ca^{2+} and Zn^{2+} in MuSCs during muscle regeneration but supplementation of Ca^{2+} and Zn^{2+} does not rescue the *Trpm7* cKO MuSC phenotype.

(**A** and **B**) Ca^{2+} measurements in MuSCs using Cal-520, AM. (**A**) Representative fluorescent images of Cal-520-incorporated MuSCs. Left: MuSCs isolated from injured muscle. Right: MuSCs from the injured muscle three days after muscle injury. (**B**) Quantification of mean fluorescence intensity of MuSCs isolated at the indicated time points after CTX injection.

(**C** and **D**) Cytosolic Ca^{2+} measurements of MuSCs isolated 3 days after CTX injury. (**C**) Representative fluorescent images of Cal-520-incorporated MuSCs from control and cKO. (**D**) Left: Representative histograms of flow cytometric data using Cal-520. Light grey: unstained MuSCs, dark grey: con, and red: cKO MuSCs. Right: Quantification of increase in cytosolic Ca^{2+}.

(**E** and **F**) Zn^{2+} measurements in MuSCs using FluoZin-3, AM. (**E**) Representative fluorescent images of FluoZin-3 -incorporated MuSCs. Left: MuSCs isolated from injured muscle. Right: MuSCs from the injured muscle three days after muscle injury. (**F**) Quantification of mean fluorescence intensity of MuSCs isolated at the indicated time points after CTX injection.

(**G** and **H**) Cytosolic Zn^{2+} measurements of MuSCs isolated 3 days after CTX injury. (**G**) Representative fluorescent images of FluoZin-3-incorporated MuSCs from con and cKO. (**H**) Left: Representative histograms of flow cytometric data using FluoZin-3. Light grey: unstained MuSCs, dark grey: con, and red: cKO MuSCs. Right: Quantification of increase in cytosolic Zn^{2+}.

(**I**) EdU assay on MuSCs cultured in growth medium supplemented with 10 mM Mg^{2+}, 5 mM Ca^{2+} and 10 μM Zn^{2+} for 3 days. EdU was incorporated into MuSCs for 3 h prior to fixation. Left panels: Representative images of EdU assays. Right: Quantification of EdU-positive MuSCs. (> 500 cells from N = 3 mice per condition)

Scale bars: 10 μm in (A), (C), (D), (F) and 100 μm in (G)).

Supplementary Table 1. Antibody list

Antibodies	Host	Clonality	Usage	Dilution	Catalog number	Supplier
anti-PAX7 antibody	mouse	Monoclonal	Immunocytochemistry<br>Immunohistochemistry	IC(1:500)<br>IHC(1:100)	Pax7-c	DSHB
anti-MyoD antibody	mouse	Monoclonal	Immunocytochemistry	1:200	MA512902	Thermo
anti-MyoD antibody	rabbit	Polyclonal	Immunocytochemistry	1:1000	sc-304	Santa Cruz
anti-Myogenin antibody	mouse	Monoclonal	Immunocytochemistry	1:500	F5D	DSHB
anti-Ki67 antibody	rabbit	Polyclonal	Immunocytochemistry	1:500	ab15580	Abcam
anti-Myosin 4 antibody (MF20)	mouse	Monoclonal	Immunocytochemistry	1:500	14-6503-82	eBioscience
anti-embryonic myosin heavy chain	mouse	Monoclonal	Immunocytochemistry	1:25	F1.652	DSHB
anti-Laminin	rabbit	Polyclonal	Immunohistochemistry	1:500	L9393	Sigma
anti-Collagen Ⅰ	goat	Polyclonal	Immunohistochemistry	1:500	1310-01	SouthernBiotech
anti-Human M-Cadherin	sheep	Polyclonal	Immunohistochemistry	1:500	AF4096	R&D SYSTEMS
anti-GFP mAb-Alexa Fluor 488	rat	Monoclonal	Immunohistochemistry	1:2500	D153-A48	MBL
anti-GFP antibody	goat	Polyclonal	Immunocytochemistry	1:500	AB0020-200	SICGEN
anti-TRPM7	rabbit	Polyclonal	Immunohistochemistry	1:2000	Homemade	Mori laboratory
anti-Phospho Akt (Ser473)	rabbit	Polyclonal	Immunocytochemistry	1:200	#4060	Cell Signaling Technology
anti- Akt	rabbit	Polyclonal	Immunocytochemistry	1:200	#9272	Cell Signaling Technology
anti-Phospho S6 (Ser235/236)	rabbit	Polyclonal	Immunocytochemistry	1:200	#4858	Cell Signaling Technology
anti-S6	rabbit	Polyclonal	Immunocytochemistry	1:200	#2217	Cell Signaling Technology
anti-Phospho 4E-BP (Thr37/46)	rabbit	Polyclonal	Immunocytochemistry	1:200	#2855	Cell Signaling Technology
anti-4E-BP	rabbit	Polyclonal	Immunocytochemistry	1:200	#9452	Cell Signaling Technology
anti-Phospho ERK	rabbit	Polyclonal	Immunocytochemistry	1:200	#9101	Cell Signaling Technology
anti-CyclinD1	rabbit	Polyclonal	Immunocytochemistry	1:250	ab134175	Abcam
anti-CDK4	rabbit	Polyclonal	Immunocytochemistry	1:200	11026-1-AP	Proteintech
anti-Phopho Rb	rabbit	Polyclonal	Immunocytochemistry	1:500	#8516	Cell Signaling Technology
anti-Rb	mouse	Monoclonal	Immunocytochemistry	1:200	#9309	Cell Signaling Technology
anti-p21	rabbit	Polyclonal	Immunocytochemistry	1:500	#2947	Cell Signaling Technology
anti-FOS	mouse	Monoclonal	Immunocytochemistry	1:50	sc-166940	Santa Cruz Biotechnology
anti-c-Jun	rabbit	Polyclonal	Immunocytochemistry	1:500	#9165	Cell Signaling Technology
anti-mouse IgG, Alexa Fluor 488	donkey	Polyclonal	Immunocytochemistry<br>Immunohistochemistry	1:500	A-21202	Thermo
anti-mouse IgG, Alexa Fluor 555	goat	Polyclonal	Immunocytochemistry	1:500	A-21424	Thermo
anti-rabbit IgG, Alexa Fluor 488	goat	Polyclonal	Immunocytochemistry	1:500	A-11008	Thermo
anti-rabbit IgG, Alexa Fluor 555	goat	Polyclonal	Immunocytochemistry<br>Immunohistochemistry	1:500	A-21429	Thermo
anti-goat IgG, Alexa Fluor 488	donkey	Polyclonal	Immunocytochemistry<br>Immunohistochemistry	1:500	A-11055	Thermo
anti-sheep IgG, Alexa Fluor 555	donkey	Polyclonal	Immunohistochemistry	1:500	A-21436	Thermo

Supplementary Table 2. Oligonucleotide sequences

qPCR

Target		Sequence
Trpm7	F	ATTTGCCCGTGATACCCCAG
	R	CAGCTTTCTGCTTGCACCG
MyoD	F	ACTGCTCTGATGGCATGATG
	R	CACAGCCGCACTCTTCCC
Cdk4	F	GGCCCTCAAGAGTGTGAGAG
	R	CATCAGCCGTACAACATTGG
18s	F	TTCTGGCCAACGGTCTAGACAAC
	R	CCAGTGGTCTTGGTGTGCTGA

Genotyping

Target		Sequence
Trpm7 ^{flox}	F	TACATGTCTCTAGTCCAGCTTC
	R	TACAAGGGCAAACTTGGAAGAC
Pax7 ^{CreERT2}	F	TACCAGAGGCAACAAACAGG
	R	CAAAGGTGGCTAAGGTGGAG
Rosa26-YFP	F	AGGGCGAGGAGCTGTTCA
	R	TGAAGTCGATGCCCTTCAG
TRPM7KR	F	ATGGGAGGTGGTTTACGA
	R	GGGAAGGGCCTTATCAATATG
